# Upregulation of ENAH by a PI3K/AKT/β-catenin cascade promotes oral cancer cell migration and growth via an ITGB5/Src axis

**DOI:** 10.1186/s11658-024-00651-0

**Published:** 2024-11-07

**Authors:** Xiu-Ya Chan, Kai-Ping Chang, Chia-Yu Yang, Chiao-Rou Liu, Chu-Mi Hung, Chun-Chueh Huang, Hao-Ping Liu, Chih-Ching Wu

**Affiliations:** 1 Graduate Institute of Biomedical Sciences, College of Medicine, Chang Gung University, Taoyuan, Taiwan; 2 Molecular Medicine Research Center, Chang Gung University, Taoyuan, Taiwan; 3 Department of Otolaryngology-Head and Neck Surgery, Linkou Chang Gung Memorial Hospital, Taoyuan, Taiwan; 4 Department of Microbiology and Immunology, College of Medicine, Chang Gung University, Taoyuan, Taiwan; 5 Department of Medical Biotechnology and Laboratory Science, College of Medicine, Chang Gung University, Taoyuan, Taiwan; 6 Department of Veterinary Medicine, College of Veterinary Medicine, National Chung Hsing University, Taichung, Taiwan

**Keywords:** Protein-enabled homolog (ENAH), Oral cavity squamous cell carcinoma (OSCC), Patient-derived xenograft (PDX), Integrin β5 (ITGB5), Proteomics, GSK3β/β-catenin signaling

## Abstract

**Background:**

Oral cancer accounts for 2% of cancer-related deaths globally, with over 90% of cases being oral cavity squamous cell carcinomas (OSCCs). Approximately 50% of patients with OSCC succumb to the disease within 5 years, primarily due to the advanced stage at which it is typically diagnosed. This underscores an urgent need to identify proteins related to OSCC progression to develop effective diagnostic and therapeutic strategies.

**Methods:**

To identify OSCC progression-related proteins, we conducted integrated proteome and transcriptome analyses on cancer tissues from patients and patient-derived xenograft (PDX) model mice. We investigated the role of protein-enabled homolog (ENAH), identified as an OSCC progression-associated protein, through proliferation, transwell migration, and invasion assays in OSCC cells. The mechanisms underlying ENAH-mediated functions were elucidated using gene knockdown and ectopic expression techniques in OSCC cells.

**Results:**

ENAH was identified as a candidate associated with OSCC progression based on integrated analyses, which showed increased ENAH levels in primary OSCC tissues compared with adjacent noncancerous counterparts, and sustained overexpression in the cancer tissues of PDX models. We confirmed that level of ENAH is increased in OSCC tissues and that its elevated expression correlates with poorer survival rates in patients with OSCC. Furthermore, the upregulation of ENAH in OSCC cells results from the activation of the GSK3β/β-catenin axis by the EGFR/PI3K/AKT cascade. ENAH expression enhances cell proliferation and mobility by upregulating integrin β5 in oral cancer cells.

**Conclusions:**

The upregulation of ENAH through a PI3K/AKT/β-catenin signaling cascade enhances oral cancer cell migration and growth via the ITGB5/Src axis. These findings offer a new interpretation of the ENAH function in the OSCC progression and provide crucial information for developing new OSCC treatment strategies.

**Supplementary Information:**

The online version contains supplementary material available at 10.1186/s11658-024-00651-0.

## Introduction

Oral cancer is a type of head and neck squamous cell carcinoma (HNSCC), affects more than half a million people worldwide each year [1], and was ranked as the sixth highest cause of cancer deaths in Taiwan for a decade [2]. OSCC accounts for more than 90% cases of oral cancers [3]. In spite of advances in OSCC treatment over the past 10 years, the 5-year survival of patients with OSCC was not ameliorated and is still only about 50% [4]. This poor outcome predominantly stems from the metastasis and progression of the disease, highlighting the urgent need to unravel the mechanisms underlying OSCC metastasis and progression to develop effective treatments [5].

To date, only a few molecules have been approved with potential for OSCC treatment. The PI3K/AKT pathway is a downstream signal transduction cascade of epidermal growth factor receptor (EGFR) and is active in more than 90% of HNSCC cases [6]. Many efforts have been made to develop more effective and safer inhibitors of PI3K/AKT and EGFR signaling pathways, however, clinical evaluation of PI3K inhibitors in HNSCC is currently in early phase of clinical trials [7]. The Wnt/β-catenin signaling plays a vital role in tumorigenesis and progression of HNSCC. Thus, this signaling cascade is a potential therapeutic target for HNSCC [8]. LGK974 is an inhibitor of porcupine, an enzyme promotes palmitoylation of Wnt and thereby involves in activation of Wnt signals, and is currently evaluated in a phase II clinical trial of HNSCC (NCT02649530) [9]. Moreover, ICG-001, a small molecule that inhibits the interaction between β-catenin and CREB binding protein, is currently testified in phase I clinical trials in patients with HNSCC [8].

Cancer cell lines have been widely used in cancer research. However, due to genetic variations and the absence of a tumor microenvironment (TME) that includes various cellular components, cell lines may not fully capture the intricate interplay within cancer tissues. Patient-derived xenografts (PDXs) involve transplanting small pieces of tumor tissue from cancer patients into immunodeficient mice, allowing the cancers to grow and be surgically transferred into other mice. PDXs are considered ideal and clinically relevant models for studying cancer progression, as they typically retain the histopathological and cellular structures of the original tumors [10]. To gain a deeper understanding of oral cancers, Li et al. characterized protein profiles in patient-derived xenograft (PDX) models of HNSCCs using a reverse-phase protein array [11]. Similarly, we established OSCC PDX models and identified potential therapeutic targets through transcriptome analysis [12]. However, the proteome of cancer tissues from OSCC PDX models has yet to be explored using mass spectrometry.

In this study, PDX mouse models were generated using tumor tissues from five patients with OSCC. The proteomes of tissues from adjacent noncancerous epithelia, primary OSCCs, and first- and second-generation PDX tumors were simultaneously profiled using isobaric tags for relative and absolute quantitation (iTRAQ) in conjunction with tandem mass spectrometry (MS). Among the dysregulated proteins in cancer tissues, protein-enabled homolog (ENAH) was selected for further investigation due to its elevated levels in primary cancer tissues compared to adjacent noncancerous tissues and its persistent overexpression in PDX tumor tissues. Immunohistochemistry confirmed that ENAH level is elevated in OSCC tissues, and its upregulation is correlated with poorer prognosis. Moreover, ENAH upregulation is driven by the activation of the GSK3β/β-catenin axis via the EGFR/PI3K/AKT pathway in OSCC cells. ENAH expression enhances cell proliferation and mobility through the upregulation of integrin β5 in OSCC cells. In conclusion, the findings in this study give a new insight into the ENAH role in OSCC progression and offer valuable information for developing new OSCC treatment approaches.

## Materials and methods

### Patient populations and clinical specimens

To establish PDX model, tumor tissues from patients with OSCC were collected before treatment at Linkou Chang Gung Memorial Hospital (CGMH), Taoyuan, Taiwan, between 2017 and 2020. This research was approved by the institutional review board (IRB) of Linkou CGMH and conducted following the Declaration of Helsinki (approval no. 201800700B0 and 102-5685A3). All participants provided informed consent through an IRB-approved form before sample collection. Oral mucosal pathology was initially screened via conventional oral examination and confirmed by biopsy. Patients with OSCC received routine check-ups following standard protocols. For immunohistochemistry (IHC) of ENAH, tumor tissues from 304 patients with OSCC collected from 2003 to 2008 were formalin fixed and paraffin embedded at the time of specimen collection. The clinical features and patient information for PDX establishment and IHC are detailed in Supplementary Tables S1 and S7, respectively.

### PDX model establishment

To establish first-generation PDX mice (P1), small pieces of cancer tissue (50–100 mg) from five patients with OSCC (Supplementary Table S1) were subcutaneously inoculated into the left flanks of NOD.Cg-*Prkdc*^scid^ *Il2rg*^*tm1Wjl*^/SzJ (NSG) mice (The Jackson Laboratory). Animal care and experimental procedures were complied with the guidelines of the Institutional Animal Care and Use Committee, Chang Gung University, Taoyuan, Taiwan, and conducted following the Basel Declaration (approval no: CGU106-114 and CGU107-074). Once the P1 tumors reached approximately 1–2 cm^3^ in volume, they were used to establish second-generation PDX (P2) models in NSG mice [10]. The details of PDX model establishment were included in Supplementary Materials and Methods.

### Tryptic digestion of tissue proteins and iTRAQ labeling

Tissue pieces (approximately 3 × 3 × 3 mm^3^) of cancerous and adjacent noncancerous counterparts were homogenized and digested with trypsin as delineated in Supplementary Materials and Methods. Tissues from adjacent noncancerous epithelia (N), primary tumors (T), and tumors from first (P1) and second (P2) generation PDX mice were comparatively analyzed. To profile the tissue proteomes with iTRAQ reagents, the tryptic peptides from the N, T, P1, and P2 samples of each patient were conjugated with isobaric tags 114, 115, 116, and 117 (AB Sciex, Foster City, CA, USA), respectively. The iTRAQ-tagged samples from the same patients were combined and desalted as depicted in Supplementary Materials and Methods.

### Peptide fractionation and liquid chromatography (LC)–MS/MS analysis

Peptide separation was performed with a two-dimensional LC (UltiMate™ 3000 RSLCnano System; Thermo Fisher Scientific, San Jose, CA, USA) as illustrated in Supplementary Materials and Methods [13]. The LC was online connected with a tandem MS (LTQ-Orbitrap Elite mass spectrometer; Thermo Fisher Scientific), which was controlled by Xcalibur software (version 2.2 SP1.48, Thermo Fisher Scientific). Methods for acquisition of MS and MS/MS data were described in Supplementary Materials and Methods.

### Peptide-spectrum matching and iTRAQ-based quantification

For peptide-spectrum matching, MS/MS spectra were searched using Mascot (version 2.2.0; Matrix Science, London, UK) against the Swiss-Prot human protein database (released in March 2023) in Proteome Discoverer software (version 1.4.1.14; Thermo Fisher Scientific), as described in Supplementary Materials and Methods. The peptide confidence criteria included a peptide length > 7 amino acids, peptide confidence *p* value < 0.01, and at least two peptide-spectrum matches per protein. Keratins were excluded from the list of identified proteins. For protein quantification, iTRAQ data were extracted from Proteome Discoverer to Microsoft Excel. Proteins with at least two quantifiable spectra were considered quantifiable proteins. Each protein ratio was log_2_-transformed and normalized with the median log_2_ ratio of all proteins. The standard deviation (SD) and mean of all protein log_2_ ratios were calculated. Proteins with log_2_ ratios exceeding the mean ratio plus one SD were considered overexpressed, while those with log_2_ ratios falling below the mean ratio minus one SD were regarded as underexpressed.

### Bioinformatics analysis

Proteins quantified in at least four iTRAQ analyses of OSCC tissues were selected to perform hierarchical clustering (HCL) analysis and principal component analysis (PCA) using the Partek Genomics Suite (Partek Inc., St. Louis, MO, USA). Functional enrichment analysis of dysregulated proteins in OSCC tissues was conducted using GO term and biological pathway analyses through the STRING database (version 11.5). HCL, PCA, and data visualization of the enrichment analyses were described in Supplementary Materials and Methods. The GEPIA2 website (http://gepia2.cancer-pku.cn/#index) was utilized to determine correlations between expressions of ENAH and integrin subunits in the TCGA HNSCC dataset.

### IHC and scoring of tissue ENAH

Tissue ENAH expression was assessed using an antibody against ENAH (1:150 dilution; cat. no. 26421–1-AP; Proteintech, Chicago, IL, USA). IHC and scoring of tissue ENAH were conducted as described in Supplementary Materials and Methods.

### Cell culture and transfection

OSCC cell lines, SCC4 and OECM-1, were grown in media supplied with 10% fetal bovine serum (FBS; Avantor, Hsinchu, Taiwan) and 1% penicillin/streptomycin solution (Gibco, Waltham, MA, USA). OECM-1 cells were maintained in RPMI-1640 medium (Gibco), while SCC4 cells were grown in DMEM/F-12 medium (Gibco) containing 0.04% hydrocortisone (Sigma-Aldrich). The OSCC cells were cultured in a cell culture incubator with 5% CO_2_ at 37 °C.

For mechanistic studies, the EGFR signaling pathway in OSCC cells was regulated with epidermal growth factor (EGF; cat. no. 324831; Merck, Taipei, Taiwan) and cetuximab (erbitux; Merck). PI3K activity was inhibited using GDC0941 (cat. no. 11600; Cayman Chemical, Ann Arbor, MI, USA) and GSK1059615 (cat. no. 11569; Cayman Chemical).

To inhibit ENAH expression, ENAH Smart Pool siRNA (cat. no. L-021932-00-0010; Dharmacon, Lafayette, CO, USA) was transfected into cells using Lipofectamine RNAiMAX (cat. no. 13778150; Invitrogen, Grand Island, NY, USA). ON-TARGETplus non-targeting control siRNA (cat. no. D001810-10-20; Dharmacon) served as the control siRNA.

For overexpression studies in OSCC cells, cDNAs of the coding sequences for ENAH, β-catenin, and integrin β5 (ITGB5) were cloned and inserted into the pcDNA3.1C plasmid. Plasmid transfection was conducted using the TransIT-X2 Dynamic Delivery System (cat. no. MIR 6000; Mirus Bio, Madison, WI, USA) according to the instruction of manufacturer.

### MTT cell proliferation assay

SCC4 and OECM-1 cells were reseeded in 24-well plates post 24 h of transfection. At the indicated time points, the cells were treated with 10% MTT (cat. no. M6494; Invitrogen) at 37 °C. After 1 h, the media were discarded, and the precipitates were dissolved with dimethyl sulfoxide. After 10 min, absorbance was measured at 540 nm.

### Transwell migration and invasion assays

Transwell assays were carried out using Transwell Polyester Membrane Inserts (cat. no. 3464; Corning, Taipei, Taiwan). OSCC cells (3.5 × 10^4^ and 5 × 10^4^ cells for SCC4 and OECM-1, respectively) were seeded into transwell chambers for migration assays. For invasion assay, 5 × 10^4^ SCC4 cells and 7 × 10^4^ OECM-1 cells were seeded into the chambers coated with Matrigel (cat. no. 354234; Corning). Culture media containing 30% FBS (700 μL) were put in the lower chamber. OSCC cells suspended in 200 μL of culture media containing 0.5% FBS were added into the upper chamber. After 18 h, the OSCC cells that passed through the chambers were treated with methanol for 20 min, stained using Giemsa’s staining solution for 1 h, and counted in 10 microscope fields of view at 200 × magnification.

### Quantitative real-time PCR (qRT-PCR)

Total RNA of OSCC cells was extracted using a TOOLSmart RNA Extractor (cat. no. DPT-BD24; Biotools, New Taipei City, Taiwan). cDNA was synthesized using a TOOLSQuant II Fast RT Kit (cat. no. KRT-BA06-2; Biotools). qRT-PCR was conducted using TOOLS 2xSYBR qPCR Mix Kit (cat. no. FPT-BB05; Biotools). qRT-PCR primer sets used in this study are listed in Supplementary Table S2.

### Western blot

The protein of interest was detected with immunoblotting as detailed in Supplementary Materials and Methods. Primary antibodies used for immunoblotting included AKT antibody (1:3000; cat. no. 9272; Cell Signaling, Danvers, MA, USA), β-catenin antibody (1:60,000; cat. no. 51067-2-AP; Proteintech), non-phospho (active) β-catenin (Ser33/37/Thr41) antibody (1:1000; cat. no. 4270; Cell Signaling), ENAH antibody (1:8000; cat. no. 26421-1-AP; Proteintech), GAPDH antibody (1:3000; cat. no. sc-32233; Santa Cruz Biotechnology, Santa Cruz, CA, USA), GSK3β antibody (1:1000; cat. no. sc-9166; Santa Cruz Biotechnology), ITGB5 antibody (1:1000; cat. no. 3629; Cell Signaling), Src antibody (1:3000, cat. no. 2109; Cell Signaling), VCP antibody (1:8000; cat. no. 10736-1-AP; Proteintech), phospho-Akt (Ser473) antibody (1:1000; cat. no. 9271; Cell Signaling), phospho-GSK3α/β (Ser21/9) antibody (1:1000; cat. no. 9331; Cell Signaling), and phospho-Src (Tyr419) antibody (1:1000; cat. no. 6943; Cell Signaling).

### Statistical analysis

The comparison of ENAH levels between paired noncancerous and OSCC tissues was assessed using a paired *t*-test. For proliferation assays, a representative experiment with four replicates per group is shown, and the results of two independent replicate experiments must show similar trends. For migration, invasion, and qRT-PCR assays, all comparisons were based on data from three independent experiments. Shapiro–Wilk test was used to determine whether the data are normally distributed, as described in Supplementary Materials and Methods. Between-group differences were estimated using Student’s *t*-tests or one-way ANOVA. All analyses were performed using Prism software (version 9.0; GraphPad Software, La Jolla, CA, USA). A two-sided *p* value smaller than 0.05 is deemed to be statistically significant.

## Results

### iTRAQ-based profiling of tissues from patients with OSCC and PDX model mice

To discover OSCC-associated proteins, tissue proteome analysis was performed on primary tumors and case-matched PDXs from five patients with OSCC (#06, #29, #34, #44, and #48; Supplementary Table S1). For each patient, tissues from adjacent noncancerous epithelia (N), primary tumors (T), and tumors from first (P1)- and second (P2)-generation PDX mice were comparatively analyzed using an iTRAQ-based 2D-LC–MS/MS approach.

We identified an average of 4656 proteins per patient group (ranging from 4259 to 5358 proteins) and quantified an average of 4524 proteins (ranging from 4143 to 5194 proteins) for each group. A total of 6315 proteins were quantifiable, and the levels of 2914 (46.1%), 826 (13.1%), 710 (11.2%), 751 (11.9%), and 1114 (17.6%) proteins were detected in all, four, three, two, and one of the five groups, respectively (Supplementary Fig. S1). Detailed information regarding tissue protein detection and quantitation is available in Supplementary Tables S3 and S4.

We subsequently determined whether the tumors originating from the PDX in mice retained the characteristics of primary OSCC. We examined the correlations of protein abundances between the T, P1, and P2 tissues in each patient group. Supplementary Fig. S2A revealed a significant positive association between protein levels of the T group and those of the case-matched P1 group (*r* = 0.516–0.743; *p* < 0.0001). The correlation between protein expression in the P1 and P2 groups (*r* = 0.654–0.898; *p* < 0.0001) was stronger than that between the T and P1 groups (Supplementary Fig. S2A).

We then evaluated differences in protein landscapes among tumor tissue samples using PCA and unsupervised HCL analysis. As shown in Supplementary Fig. S2B and S2C, the proteome profiles of the T, P1, and P2 tissues showed a stronger correlation within patient groups than between them. Notably, the proteome profiles from T, P1, and P2 tissues clustered together based on the patient groups (Supplementary Fig. S2C). These results suggest that the protein expression signature of primary OSCCs is preserved in PDX tumors.

### Integrated omics analyses revealed protein candidates involved in OSCC progression

To identify proteins with altered expression levels in OSCC tissues, the means and SDs of the iTRAQ ratios for all proteins in each comparison were acquired (Table 1). Proteins with ratios exceeding the mean plus one SD were classified as overexpressed, while proteins with ratios below the mean minus one SD were classified as underexpressed. Compared with the adjacent noncancerous (N) group, average counts of 439, 430, and 439 proteins were classified as overexpressed in the tumor (T), P1, and P2 tissues, respectively. Additionally, there were slightly more proteins with reduced expression in each cancer tissue, with average counts of 504, 513, and 514 proteins downregulated in the T, P1, and P2 tissues, respectively (Table 1 and Supplementary Table S4).

**Table 1:** Number of proteins quantified with iTRAQ analysis in each patient group

OSCC ID	#06	#29	#34	#44	#48	Average
No. of protein in iTRAQ analysis^a^						
Identification	4259	4663	3883	5115	5358	4656
Quantification	4143	4545	3788	4950	5194	4524
Mean/SD of all protein ratio in iTRAQ analysis^b^						
T/N^c^	−0.146/ 1.018	−0.072/ 0.819	−0.182/ 0.957	−0.064/ 0.875	−0.091/ 0.772	−0.111/ 0.888
P1/N^c^	−0.053/ 1.165	−0.189/ 1.009	−0.258/ 1.174	−0.081/ 0.861	−0.145/ 0.938	−0.145/ 1.029
P2/N^c^	−0.177/ 1.060	−0.125/ 0.952	−0.183/ 1.036	−0.059/ 0.890	−0.084/ 0.945	−0.126/ 0.977
No. of proteins with increased/reduced levels in cancer tissue^d^						
T/N	437/517	445/472	321/485	453/521	537/525	439/504
P1/N	600/588	370/512	309/482	441/479	432/504	430/513
P2/N	387/499	418/502	370/512	503/541	515/515	439/514

^a^ No. of proteins that were identified with at least two unique peptides or can be quantified with at least two spectra

^b^ The iTRAQ ratios of proteins were transformed into log_2_ scale. The mean and standard deviation (SD) for log_2_ iTRAQ ratios of all proteins in each comparison were acquired

^c^ N, non-cancerous tissue; T, tumor tissue; P1, first-generation of PDX tumor tissue; P2, second-generation of PDX tumor tissue

^d^ Proteins with values larger than the mean + SD were deemed overexpressed, whereas proteins with values less than the mean − SD were considered underexpressed

**Fig. 1:**
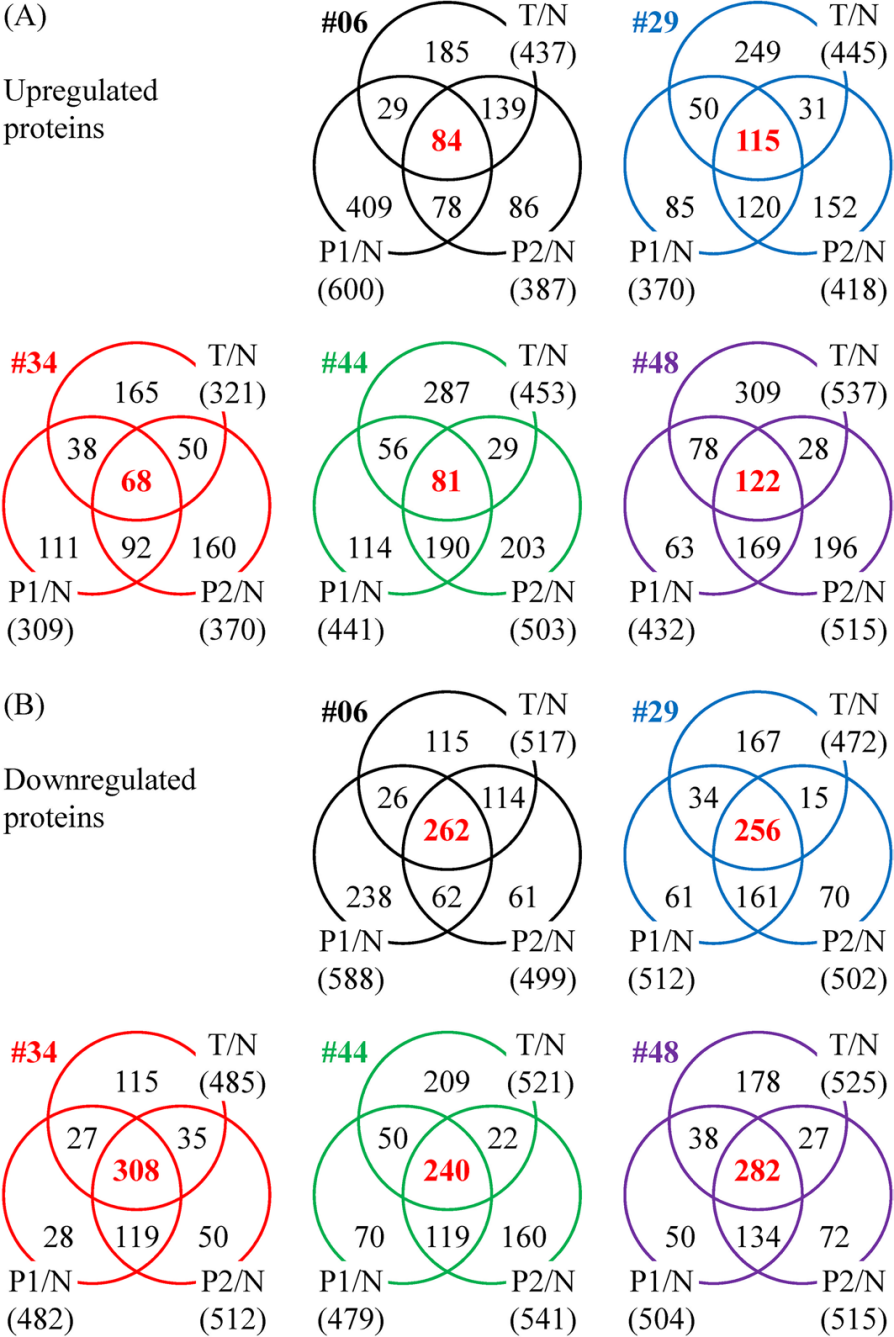
Proteome profiling of tumor tissues collected from patients with OSCC and PDX model mice. The PDX mouse models were established using tumor tissues from five patients with OSCC (#06, #29, #34, #44, and #48). The tissue proteomes of adjacent noncancerous epithelia (N), primary tumors (T), first (P1) and second (P2) generation PDX tumors were profiled using iTRAQ-based mass spectrometry. Protein ratios for T/N, P1/N, and P2/N were calculated. The means and standard deviations (SDs) for the ratios of all proteins in each comparison were determined. Proteins with ratios above the mean plus SD were considered overexpressed (**A**), while those below the mean minus SD were considered underexpressed (**B**). Venn diagrams illustrate the overlap between the differentially expressed proteins in the T, P1, and P2 groups

We conducted experiments to establish PDX models using cancer tissues from 49 patients with OSCC. To date, we have successfully generated 12 PDX models [10]. The OSCC tissues capable of developing PDX models likely harbor dysregulated proteins essential for cancer progression. Consequently, the proteins differentially expressed in the primary cancer tissues would remain dysregulated in the PDX mice's cancer tissues. To identify OSCC progression-associated proteins, we determined the proteins simultaneously dysregulated in the T, P1, and P2 tissues. According to these criteria, 84, 115, 68, 81, and 122 proteins were commonly upregulated in the three tumor tissues (T, P1, and P2) of the #06, #29, #34, #44, and #48 groups, respectively (Fig. 1A). Compared with the N tissues, 262, 256, 308, 240, and 282 proteins were downregulated in all three tumor tissues of the #06, #29, #34, #44, and #48 groups, respectively (Fig. 1B).

To minimize the impact of interindividual differences, we selected 215 proteins consistently dysregulated in at least three patient groups, including 28 overexpressed and 187 underexpressed proteins (Supplementary Table S5). To efficiently isolate progression-related candidates, we analyzed the gene expression profiles of these 215 proteins using RNA-seq on T, P1, and P2 tissues from the #06, #29, #34, and #48 groups [10, 12]. Compared with the N tissues, 18 of the 28 genes were upregulated (defined as > 2-fold change) in the T, P1, and P2 tissues of more than three patient groups. Conversely, 45 of the 187 genes were downregulated (defined as < 0.5-fold change) in the three tumor tissues of at least three patient groups (Supplementary Table S5).

### Bioinformatics analysis of proteins required for OSCC progression

**Fig. 2:**
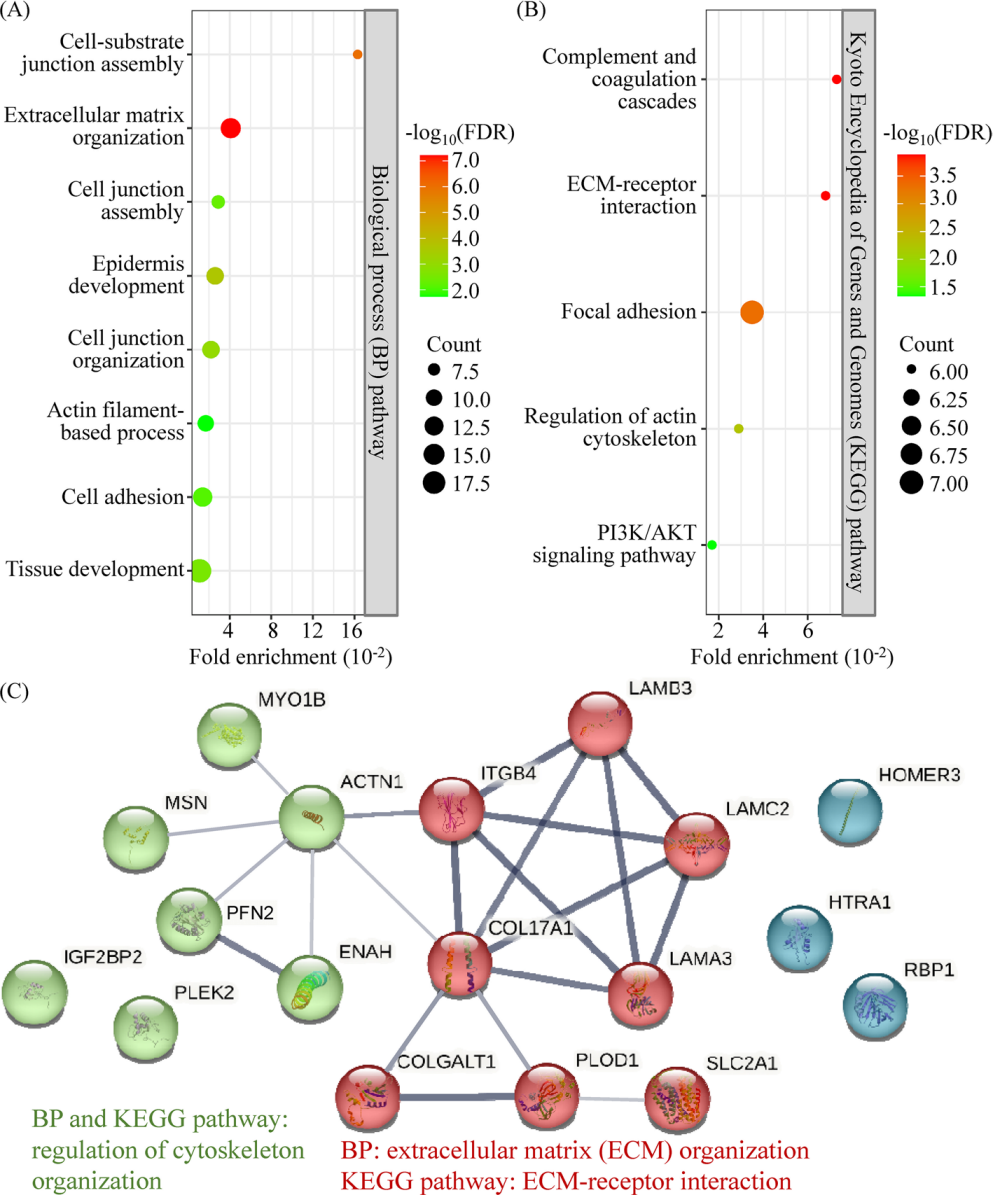
Pathway analysis of proteins involved in OSCC progression. The OSCC progression-related proteins (18 upregulated and 45 downregulated proteins; see Supplementary Table S5) were functionally annotated with STRING v11.5 software. The biological process (**A**) and KEGG pathway (**B**) analyses were conducted and visualized with online SRplot software. **C** A protein–protein interaction network of the 18 upregulated proteins was constructed, revealing 21 interaction links between the 18 proteins/nodes (indicated by solid lines). Two modules identified within the network illustrate the interactions of proteins involved in actin cytoskeleton pathways (green nodes) and ECM organization regulation (red nodes)

To uncover the biological processes and pathways involving the progression-related proteins, 63 proteins (18 upregulated and 45 downregulated proteins) were functionally annotated using the STRING database. As depicted in Fig. 2A, these proteins are closely associated with biological processes such as extracellular matrix (ECM) organization, cell junction organization/assembly, and actin filament-based process (Supplementary Table S6). Pathway analysis using the Reactome and Kyoto Encyclopedia of Genes and Genomes (KEGG) databases uncovered that the proteins are likely associated with ECM–receptor interaction, actin cytoskeleton regulation, and PI3K/AKT signaling pathways (Fig. 2B and Supplementary Table S6).

Protein–protein interaction (PPI) networks for the 18 upregulated proteins were further predicted using the STRING database, revealing 21 interaction links (Fig. 2C). Consistent with the enrichment analysis of biological processes and pathways (Fig. 2A, B), the PPI analysis identified two modules outlining interactions between proteins related to the cytoskeleton and ECM organization regulation (Fig. 2C). Overall, these results suggest that proteins involved in OSCC progression and PDX establishment play crucial roles in the regulation of ECM organization and actin cytoskeleton.

### Evaluation of ENAH as an OSCC progression-associated protein

**Fig. 3:**
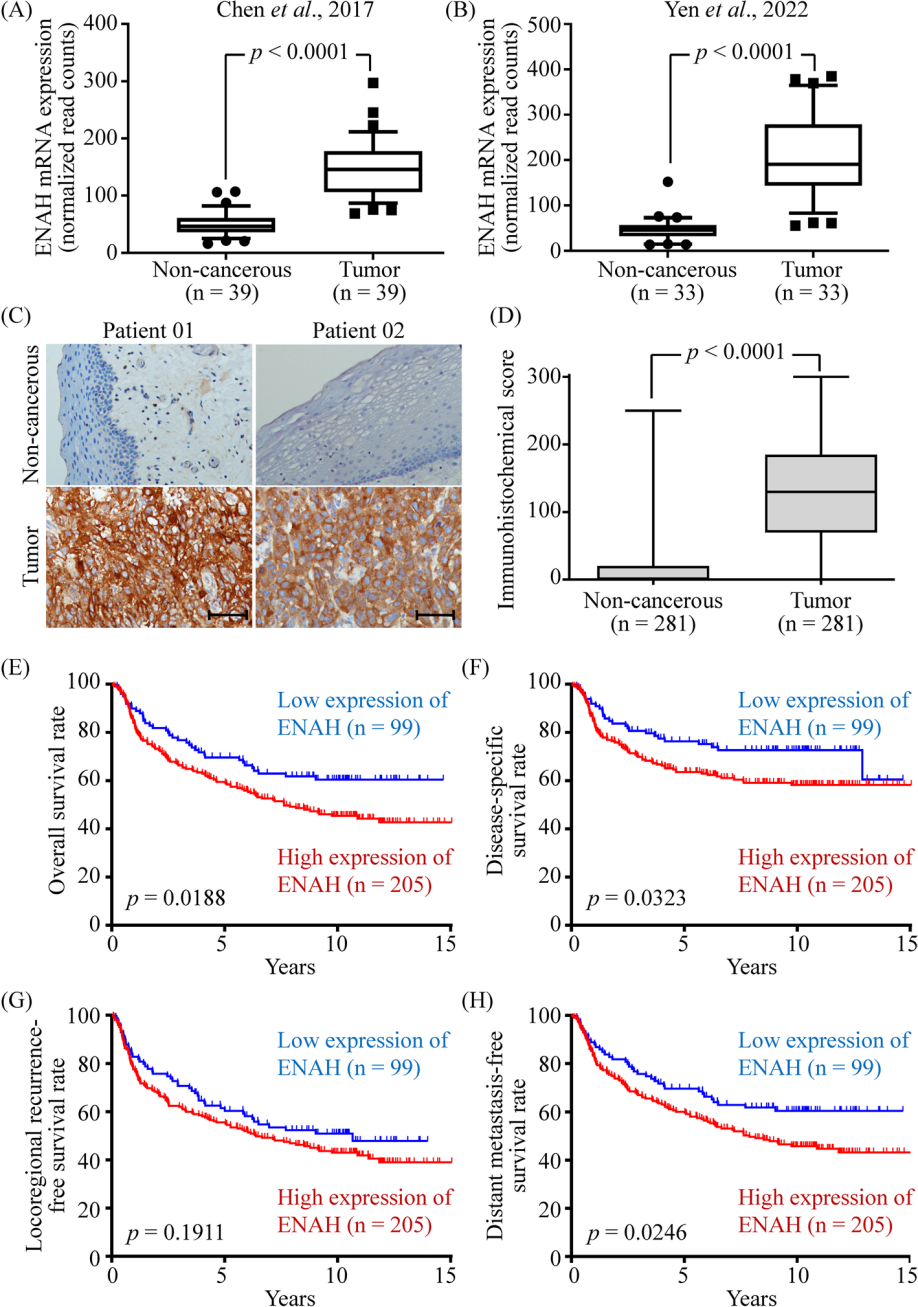
A higher level of tissue ENAH was associated with poor survival in patients with OSCC. The gene expression of ENAH in OSCC tissues was examined using RNA-seq data from Chen et al., Nat Commun. 2017, 8, 465 (**A**), and Yen et al., Front Oncol. 2022, 12, 792,297 (**B**). Differences between groups were determined using paired *t*-tests. (**C**) Representative images of ENAH IHC staining in paired adjacent noncancerous (upper panel) and OSCC (lower panel) tissues are shown. Scale bar: 100 μm. Original magnification: × 200. **D** The IHC scores of paired noncancerous and cancerous tissues are represented as box and whisker plots, where whiskers, boxes, and horizontal lines depict the distributions of the middle 90%, upper and lower quartiles, and medians of the IHC scores, respectively. Kaplan–Meier plots show the long-term overall (**E**), disease-specific (**F**), locoregional recurrence-free (**G**), and distant metastasis-free (**H**) survival of 304 patients with OSCC. The plots were stratified using the first tertile of the ENAH IHC scores, with *p* values obtained using log-rank tests

ENAH, one of the 18 upregulated proteins (Supplementary Table S5), was selected to verify its role in promoting OSCC progression, as it has been proven to participate in the formation of actin-dependent filopodia [14] and the progression of gastric cancer [15]. To confirm the upregulation of ENAH in OSCC tissues, we analyzed RNA-seq data from two independent cohorts of Taiwanese patients with OSCC [10, 16]. As shown in Figs. 3A, B, ENAH was significantly upregulated in OSCC tissues compared with noncancerous counterparts.

We further performed IHC to assess ENAH levels in biopsy specimens from 304 patients with OSCC (Supplementary Table S7). Among 304 biopsies, noncancerous epithelia were present in 281 sections. The ENAH level was significantly raised in OSCC tissues compared with paired noncancerous epithelia (Fig. 3C). The IHC score of ENAH in OSCC tissues (130.0 ± 77.03) was significantly higher than in adjacent noncancerous tissues (19.59 ± 39.07, *n* = 281, *p* < 0.001; Fig. 3D). However, in this case–control study, the ENAH level in OSCC tissues was not statistically associated with tumor size, lymphatic metastasis, overall stage, perineural invasion, cell differentiation, or lymphovascular invasion (Supplementary Table S7).

The ENAH IHC results of 304 patients with OSCC were analyzed to determine whether ENAH overexpression was associated with patient survival. As shown in Fig. 3E–H, patients with OSCC with higher ENAH expression had worse overall, disease-specific, and distant metastasis-free survival. Importantly, ENAH overexpression in OSCC tissues was a significant predictor of worse survival, as demonstrated by univariate survival analysis using Cox proportional hazards model (univariate hazard ratio = 1.551, *p* = 0.0198; Table 2). Additionally, tissue ENAH expression was identified as an independent predictor of long-term overall survival in patients with OSCC (multivariate hazard ratio = 1.761, *p* = 0.0034; Table 2). These findings indicate that ENAH is a possible progression-associated protein in OSCC.

**Table 2:** Univariate and multivariate analysis of overall survival in patients with OSCC after treatment

Variables	Univariate crude HR (95% CI)^a^	*p* value	Multivariate adjusted HR (95% CI)^a^	*p* value
Age				
> 51.25 years versus ≤ 51.25 years	1.601 (1.152–2.223)	0.0050^b^	1.374 (0.983–1.922)	0.0633
Gender				
Female versus Male	0.753 (0.465–1.218)	0.2473	0.652 (0.399–1.064)	0.0870
Overall pathological stage				
II–IV versus I	2.339 (1.616–3.385)	< 0.0001^b^	2.105 (1.431–3.097)	0.0002^b^
Perineural invasion				
Yes versus No	1.884 (1.363–2.606)	0.0001^b^	1.496 (1.060–2.113)	0.0220^b^
Cell differentiation^a^				
P-D versus W-D + M-D	2.446 (1.564–3.824)	< 0.0001^b^	2.102 (1.289–3.427)	0.0029^b^
Lymphovascular invasion				
Yes versus No	1.538 (0.831–2.844)	0.1702	0.812 (0.425–1.551)	0.5274
ENAH expression				
High versus Low	1.551 (1.072–2.243)	0.0198^b^	1.761 (1.206–2.573)	0.0034^b^

^a^ CI, confidence interval; HR, hazards ratio; W-D, well-differentiated; M-D, moderately differentiated; P-D, poorly differentiated

^b^ Statistically significant

### ENAH enhances the proliferation and migration ability of OSCC cells

**Fig. 4:**
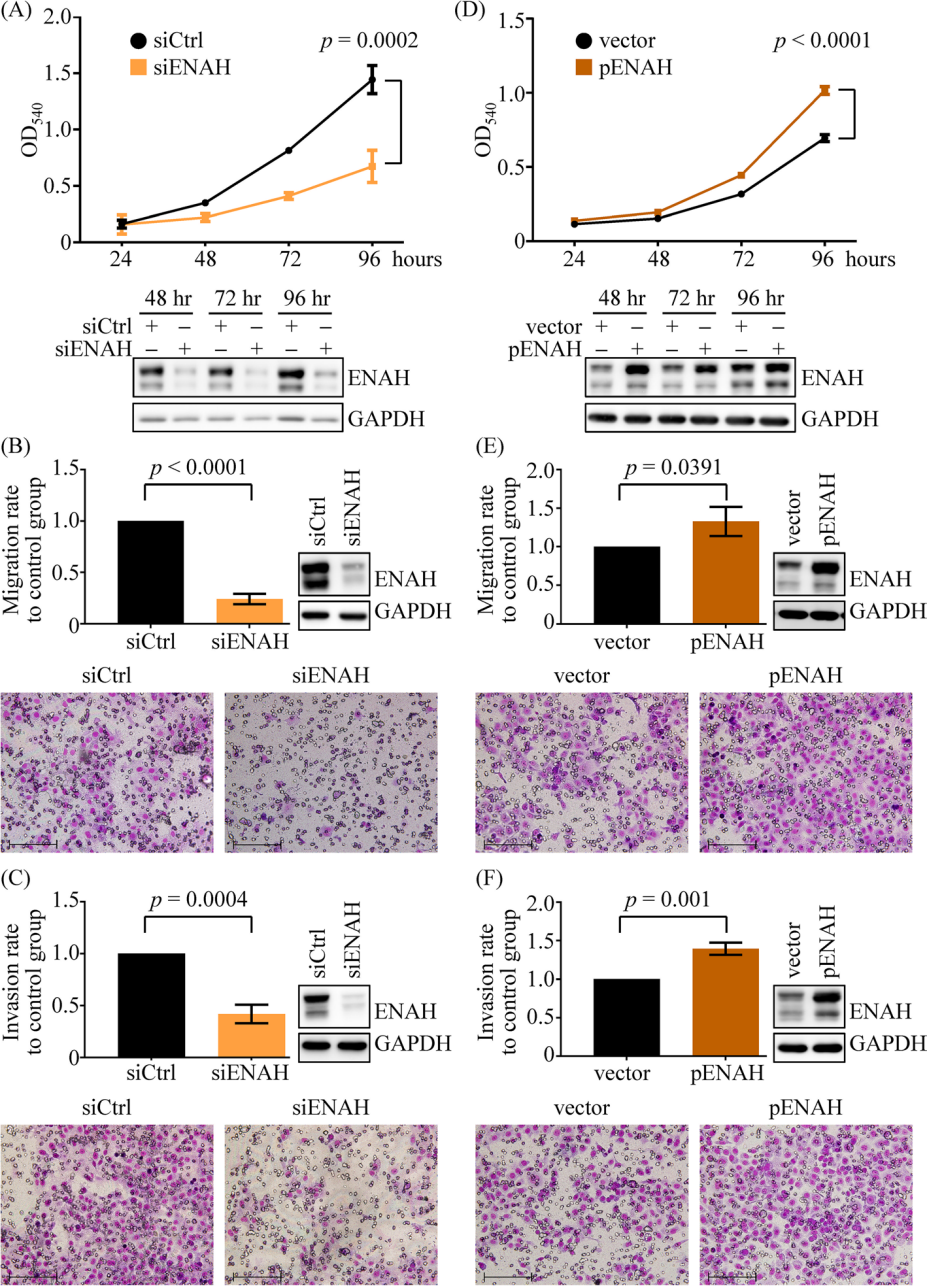
ENAH enhances the proliferation, migration, and invasion of OSCC cells. **A**–**C** MTT cell proliferation (**A**), transwell migration (**B**), and Matrigel invasion (**C**) assays were conducted with control (siCtrl) and ENAH-knockdown (siENAH) SCC4 cells. **D**–**F** The growth (**D**), migration (**E**), and invasion (**F**) abilities of SCC4 cells transfected with either the control vector (vector) or the ENAH expression plasmid (pENAH) were evaluated. The representative images of transwell migration (lower panels of **B**, **E**) and matrigel invasion (lower panels of **C**, **F**) assays were shown (original magnification, × 200; scale bar: 200 μm). The efficacy of knockdown and overexpression was confirmed by ENAH immunoblotting. For MTT assays, a representative experiment with four replicates per group is shown, and the results of two independent replicate experiments must show similar trends. For transwell assays, comparisons were based on data from three independent experiments. The *p* values were determined using Student's *t*-test

To explore function of ENAH in OSCC progression, we utilized a siRNA approach to reduce endogenous ENAH levels in two OSCC cell lines, SCC4 and OECM-1 (Supplementary Fig. S3), and subsequently assessed the impact on cell survival and mobility. MTT assays revealed that ENAH knockdown reduced the viability of SCC4 (Fig. 4A) and OECM-1 (Supplementary Fig. 4A) cells. Transwell-based experiments showed that ENAH knockdown suppressed the migratory and invasive properties of SCC4 (Fig. 4B, C) and OECM-1 (Supplementary Fig. S4B and S4C) cells. Conversely, compared with control groups, ENAH overexpression (Supplementary Fig. S3) markedly enhanced the proliferation (Fig. 4D and Supplementary Fig. S4D), migration (Fig. 4E and Supplementary Fig. S4E), and invasion (Fig. 4F and Supplementary Fig. S4F) of OSCC cells. These findings demonstrate a functional correlation between ENAH upregulation and the progression potential of OSCC.

## ENAH expression is regulated by the EGFR/PI3K/AKT signaling *axis* in OSCC cells

After establishing a functional link between ENAH and cell growth and mobility, we next identified potential upstream regulators responsible for ENAH overexpression in OSCC cells. Enrichment analysis showed that the PI3K/AKT signaling pathway is associated with OSCC progression (Fig. 2B). The PI3K/AKT and EGFR signaling pathways have been reported to play crucial roles in OSCC progression [17, 18]. Therefore, we investigated whether ENAH upregulation occurs via the EGFR and PI3K/AKT signaling pathways in OSCC cells.

**Fig. 5:**
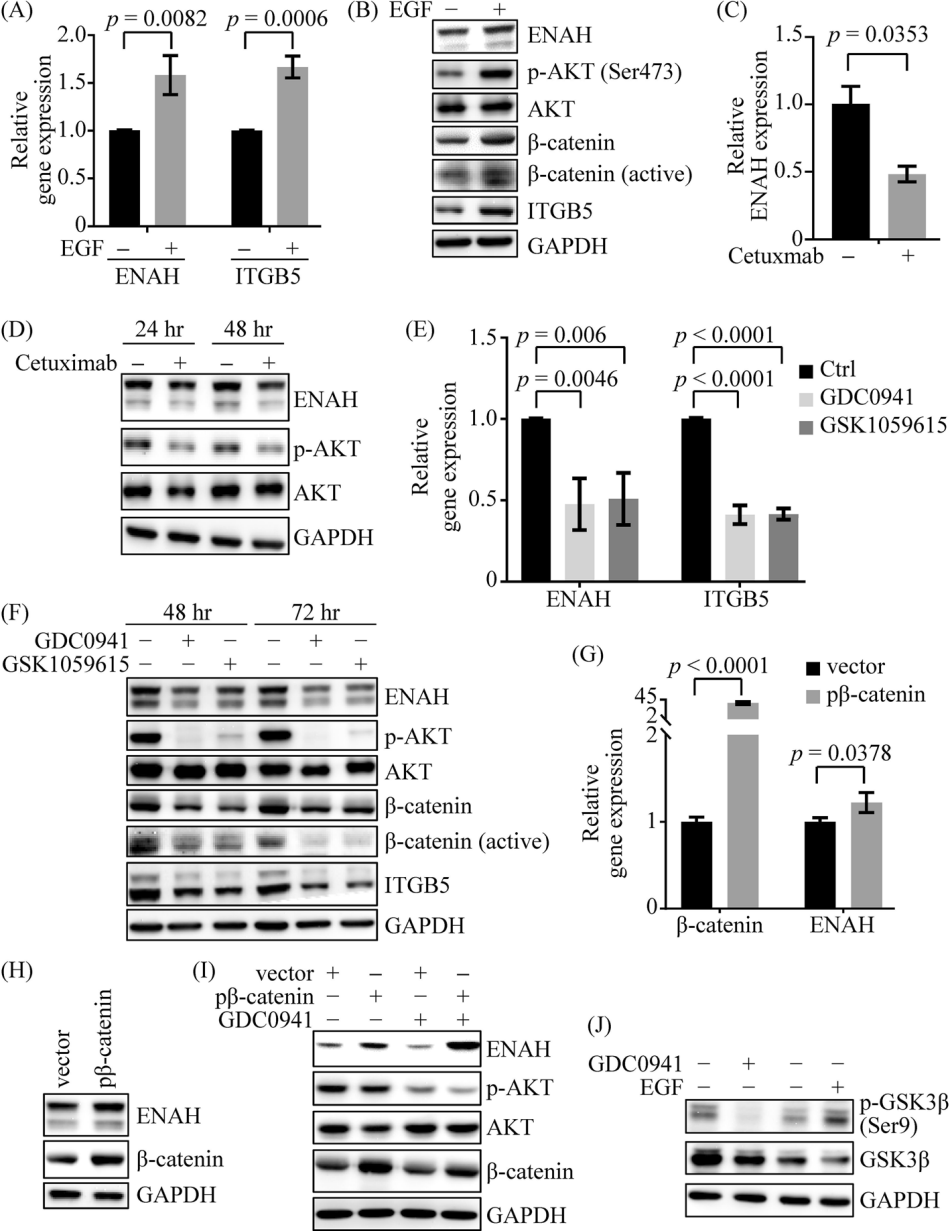
ENAH expression is regulated by the EGFR/PI3K/AKT signaling pathway in OSCC cells. After 16 h of serum starvation, SCC4 cells were treated with 40 ng/mL EGF (**A**, **B**) or 30 μg/mL cetuximab (**C**, **D**). Levels of gene expression were measured by qRT-PCR (A) and western blotting (**B**) after 12 h and 48 h of EGF treatment, respectively. After 24 h (**C**, **D**) and 48 h (**D**) of cetuximab treatment, ENAH expression was assessed by qRT-PCR (**C**) and western blot (**D**). **E**, **F** SCC4 cells were treated with PI3K inhibitors GDC0941 (9 μM) and GSK1059615 (3 μM) for 24 h for qRT-PCR (**E**) and 48–72 h for western blot (**F**) analysis. **G**, **H** SCC4 cells were transfected with the control vector (vector) or a β-catenin expression plasmid (pβ-catenin). After 48 h, qRT-PCR (**G**) and western blotting (**H**) were carried out. **I** SCC4 cells were transfected with pβ-catenin and then treated with 150 nM GDC0941. **J** The phosphorylation of GSK3β at Ser 9 (inhibition of GSK3β activity) was analyzed after treating cells with EGF (40 ng/mL) or GDC0941 (9 μM) for 2 h. Phosphorylation at Ser 9 (the 47-kDa protein band) was significantly reduced in the GDC0941-treated group and enhanced in the EGF-treated group. For qRT-PCR analysis, comparisons were based on data from three independent experiments. The *p* values were determined using Student’s *t*-test

As shown in Figs. 5A, B, EGF stimulation led to increased expression of ENAH in SCC4 cells compared with the control group. Conversely, ENAH expression decreased in SCC4 cells when ligand binding to EGFR was blocked with cetuximab (Fig. 5C, D). In addition, EGF and cetuximab treatments resulted in elevated (Fig. 5B) and reduced (Fig. 5D) levels of AKT phosphorylation, respectively.

To clarify the connection between ENAH expression and the PI3K/AKT pathway, we used PI3K inhibitors GDC0941 and GSK1059615. Both inhibitors suppressed PI3K activity, as monitored by reduced AKT phosphorylation. As shown in Figs. 5E, F, ENAH expression was lower in SCC4 cells treated with PI3K inhibitors than in control cells. Interestingly, we observed that ENAH may positively regulate the level of AKT phosphorylation in SCC4 cells (Supplementary Fig. S5A), and ENAH knockdown partially diminished EGFR-mediated AKT phosphorylation (Supplementary Fig. S5B). These findings suggest that ENAH expression is regulated by the EGFR/PI3K/AKT signaling pathway in OSCC cells.

### ENAH is upregulated through the PI3K/AKT/β-catenin axis in OSCC cells

Since EMAH has been identified as a transcriptional target of the Wnt/β-catenin pathway in hepatoma cells [19] and this pathway is frequently involved in the pathogenesis of oral cancer [17], we aimed to ascertain whether Wnt/β-catenin plays a regulatory role in ENAH expression in OSCC cells. As shown in Figs. 5G, H, β-catenin overexpression led to increased levels of ENAH in SCC4 cells compared with control cells. Moreover, the levels of total and active β-catenin were elevated in SCC4 cells with EGF stimulation (Fig. 5B) and reduced in cells with treatment of PI3K inhibitors (Fig. 5F), suggesting that the EGFR/PI3K/AKT signaling axis acts as an upstream regulatory of β-catenin.

We next investigated whether the EGFR/PI3K/AKT axis modulates ENAH expression through β-catenin signaling. ENAH expression was suppressed in SCC4 cells treated with GDC0941, and β-catenin overexpression restored the GDC0941-mediated reduction in ENAH levels (Fig. 5I). The results suggest that ENAH upregulation occurs through the EGFR/PI3K/AKT/β-catenin cascade in OSCC cells.

Inhibition of glycogen synthase kinase 3β (GSK3β) activity results in β-catenin accumulation, thus activating the β-catenin signaling pathway. Moreover, GSK3β is a downstream target of the PI3K/AKT signaling pathway [20]. Therefore, we investigated the relationship between the EGFR/PI3K/AKT/β-catenin cascade and GSK3β activity. As shown in Fig. 5J, EGF treatment inhibited GSK3β activity, as evidenced by increased phosphorylation at Ser9. Conversely, inhibition of PI3K led to reduced phosphorylation at Ser9. These findings suggest that the EGFR/PI3K/AKT signaling axis inhibits GSK3β activity, leading to β-catenin accumulation and upregulation of ENAH expression.

### ITGB5 is a downstream target of ENAH in OSCC cells

Integrins are the primary ECM receptors that establish connections between the external matrix scaffold and the internal actin cytoskeleton and play a role in processes associated with cancer progression [21]. Since progression-related proteins in OSCC are involved in ECM–receptor interactions, focal adhesion, and actin cytoskeleton regulation (Fig. 2 and Supplementary Table S6), we investigated the impact of ENAH on the expression of integrins in OSCC. Eight integrin subunits (ITGA1, ITGA3, ITGA5, ITGA6, ITGAV, ITGB1, ITGB5, and ITGB6) were selected for analysis as ENAH downstream candidates based on the observation that their expression levels were increased in OSCC tissues (T/N > 1.5 and *p* < 0.05) and exhibited positive correlations with ENAH expression in HNSCC tissues (*r* > 0.3 and *p* < 0.05; Supplementary Table S8).

**Fig. 6:**
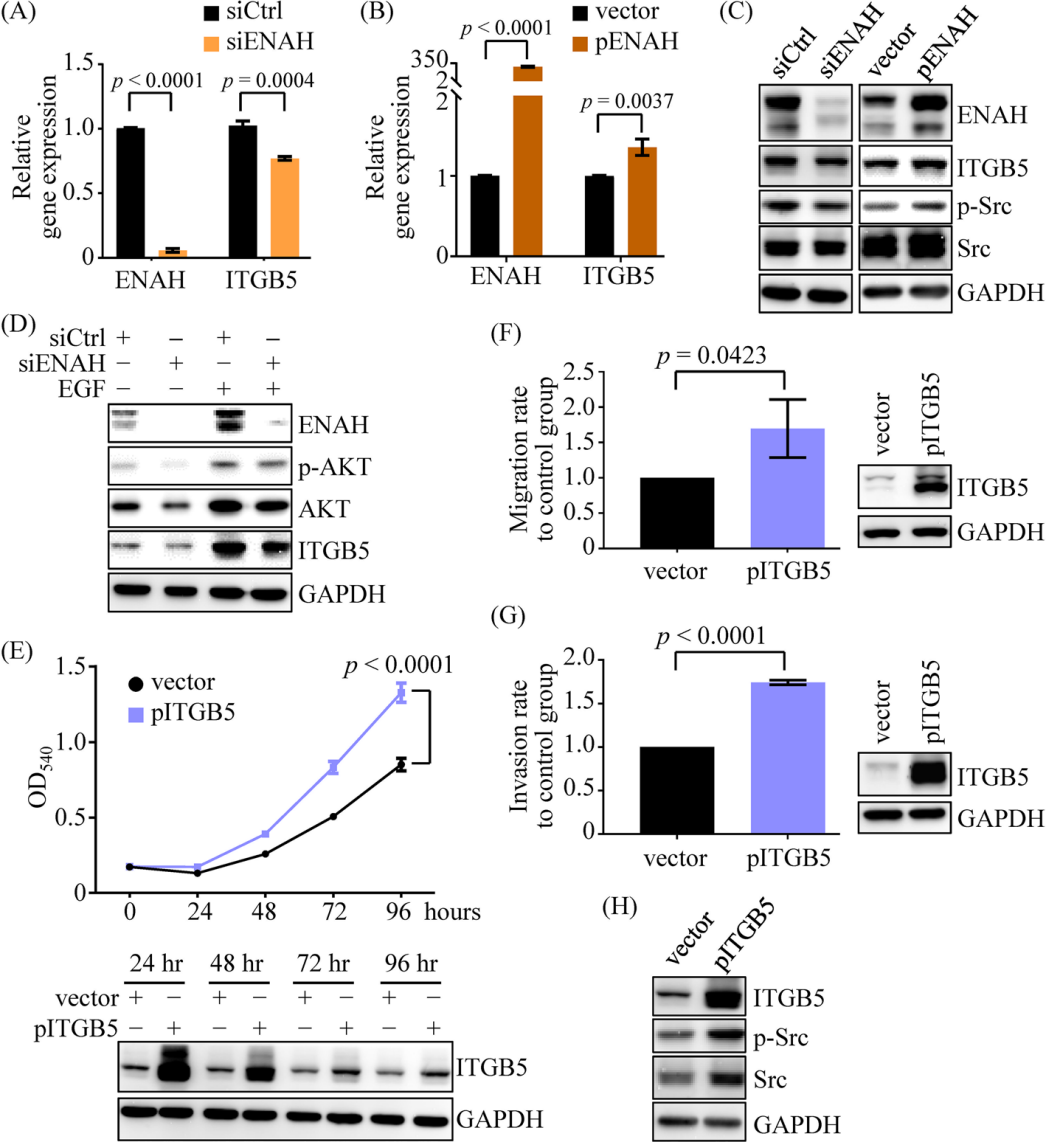
ITGB5 is a downstream target of ENAH in OSCC cells. **A**, **B** ENAH and ITGB5 expression levels were determined in SCC4 cells transfected with control siRNA (siCtrl), ENAH siRNA (siENAH), control vector (vector), or an ENAH-expression plasmid (pENAH) using qRT-PCR. **C** The levels of ITGB5 and p-Src (Tyr416) were analyzed in ENAH-knockdown and ENAH-overexpressing SCC4 cells. **D** ITGB5 protein expression in ENAH-knockdown SCC4 cells treated with or without EGF was assessed. **E**–**G** The growth (**E**), migration (**F**), and invasion (**G**) abilities of SCC4 cells transfected with the control vector (vector) or the ITGB5-expression plasmid (pITGB5) were evaluated. **H** The levels of p-Src were investigated in ITGB5-overexpressing SCC4 cells. For MTT assays, a representative experiment with four replicates per group is shown, and the results of two independent replicate experiments must show similar trends. For qRT-PCR and transwell assays, comparisons were based on data from three independent experiments. The *p* values were determined using Student’s *t*-test

qRT-PCR analysis revealed that ITGB5 expression was downregulated in ENAH-knockdown OECM-1 and SCC4 cells, whereas ENAH overexpression led to an increase in ITGB5 expression levels in both cell lines (Fig. 6A, B, Supplementary Fig. S6, S7A, and S7B). Consistent with the qRT-PCR results, the ITGB5 protein levels were reduced and elevated in the OSCC cells transfected with ENAH siRNA and ENAH expression plasmid, respectively (Fig. 6C and Supplementary Fig. S7C). These results demonstrate that ENAH modulates expression of ITGB5 in OSCC cells.

Given that ITGB5 is a downstream target of ENAH, we next determined the effect of the EGFR/PI3K/AKT/β-catenin cascade on ITGB5 expression in OSCC cells. EGF stimulation resulted in the upregulation of ITGB5 (Fig. 5A, B), and ENAH knockdown attenuated EGF-induced ITGB5 upregulation (Fig. 6D). Furthermore, treatment with a PI3K inhibitor downregulated ITGB5 (Fig. 5E, F). These results demonstrate that the EGFR/PI3K/AKT axis is an upstream signaling pathway of ENAH, which upregulates ITGB5 expression.

### ITGB5 is a downstream effector of ENAH and promotes cell growth and migration

Given the role of ENAH in promoting OSCC progression, we hypothesized that ITGB5 might mediate the proliferation and migration of OSCC cells. We verified this hypothesis by transfecting OSCC cells with an ITGB5 expression vector. Functional assays revealed that ectopic ITGB5 expression enhanced the proliferation, migration, and invasion of SCC4 (Fig. 6E–G) and OECM-1 (Supplementary Fig. S7D-S7F) cells, indicating the involvement of ITGB5 in ENAH-mediated functions in OSCC cells.

It has been reported that ITGB5 can enhance the activation of Src kinase [22], and the Src signaling pathway participates in the ITGB5-promoted migration of OSCC cells [23]. Indeed, Src activation was enhanced by ITGB5 overexpression in OSCC cells (Fig. 6H and Supplementary Fig. S7G). We examined the link between Src activation and ENAH expression. As shown in Fig. 6C and Supplementary Fig. S7C, a decrease and an increase in Src phosphorylation were observed in OSCC cells transfected with ENAH siRNA and ENAH expression vector, respectively. These results suggest that ENAH may modulate proliferation, migration, and invasion via the ITGB5/Src signaling pathway in OSCC cells.

**Fig. 7:**
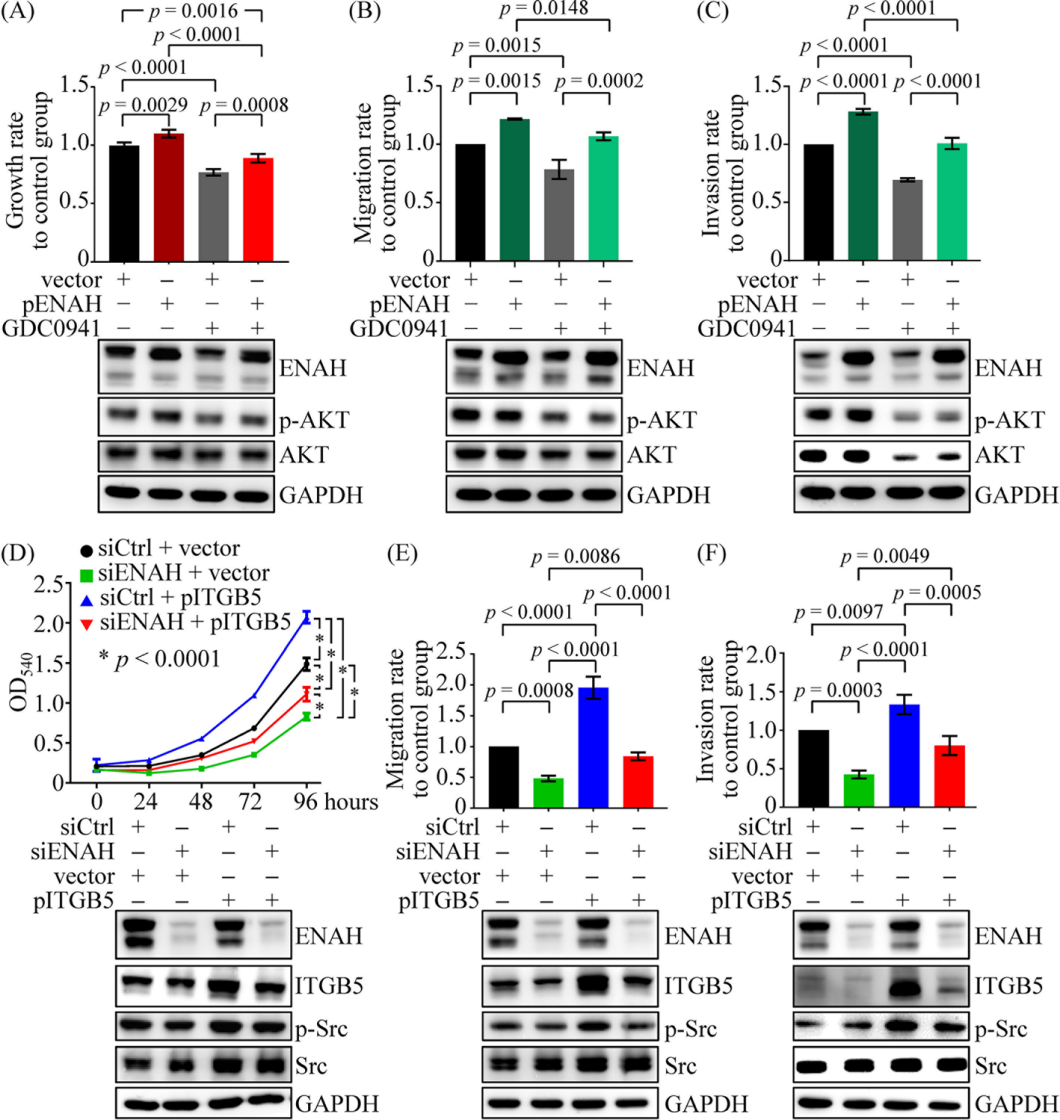
ITGB5 is a downstream effector of ENAH that promotes cell growth and migration. **A**–**C** MTT cell proliferation (**A**), transwell migration (**B**), and Matrigel invasion (**C**) assays were conducted using ENAH-overexpressing SCC4 cells with and without GDC0941 treatment (75 nM). **D**–**F** The growth (**D**), migration (**E**), and invasion (**F**) potentials were examined in ENAH-knockdown SCC4 cells with and without ITGB5 overexpression. For MTT assays, a representative experiment with four replicates per group is shown, and the results of two independent replicate experiments must show similar trends. For transwell assays, comparisons were based on data from three independent experiments. The *p* values were determined by one-way ANOVA

Furthermore, functional assays showed that ENAH overexpression reversed the inhibition of cell proliferation, migration, and invasion induced by PI3K inhibitor treatment (Fig. 7A–C). Similarly, ectopic ITGB5 expression alleviated the effects of ENAH knockdown on cell growth and mobility (Fig. 7D–F). These results confirm the involvement of ITGB5 in ENAH-regulated cell functions.

## Discussion

Due to the preservation of native cancer characteristics, PDX models have effectively addressed the limitations of discrepancies observed between cancer cell lines and translational medicine. PDX models also provide insights into the tumor microenvironment (TME) for identifying proteins involved in cancer development and progression [24]. Using a protein array, Li et al. identified proteins differentially expressed in the tumor tissues of HNSCC PDX models and suggested that proteins dysregulated in PDX tumor tissues are correlated with cancer hallmarks [11].

Consistent with the findings from HNSCC PDXs, our proteome analysis showed that the correlation between protein profiles of T and P1 in advanced-stage patients with OSCC (#29, #34, and #48; mean *r* = 0.70) was stronger than in early-stage patients with OSCC (#06 and #44; mean *r* = 0.53; Supplementary Fig. S2A). This suggests that the PDX model established with late-stage cancers more accurately preserves cancer characteristics regarding progression-related proteins. Moreover, approximately 25% of OSCC tissues have the potential to develop PDX models, implying that proteins essential for cancer progression are retained in OSCC tissues used to create PDX models.

For the first time, proteomic landscapes of primary and PDX-derived OSCC tissues were mapped comprehensively and simultaneously using iTRAQ-based mass spectrometry. The analysis identified 63 dysregulated proteins (18 upregulated and 45 downregulated) involved in processes essential for OSCC progression (Supplementary Table S6). For instance, collagen α1 (XVII) chain is upregulated in HNSCC tissues, playing a role in cell motility and adhesion and promoting HNSCC development and invasion [25]. Laminin subunit γ2 (LAMC2) contributes to the malignancy of OSCC cells and is overexpressed in OSCC due to lncRNA CASC9-mediated miR-545-3p sponging [26]. Myosin 1B (MYO1B) is also upregulated in HNSCC tissues as a result of miR-145-3p downregulation, and high MYO1B expression acts as an independent predictive factor for HNSCC patient survival [27]. Insulin-like growth factor 2 mRNA-binding protein 2 (IGF2BP2) is associated with a worse prognosis in patients with OSCC by regulating cancer-related biological pathways, such as glycolysis, epithelial-mesenchymal transition, and the cell cycle [28]. In summary, conducting proteome analysis of primary and PDX cancer tissues simultaneously is an effective strategy for identifying proteins associated with OSCC progression.

Among OSCC-related proteins identified by the mass spectrometry (Supplementary Table S5), several proteins have been associated with the PI3K/AKT and β-catenin pathways. Prolyl 4-hydroxylase subunit α2 enhances proliferation, invasion, and metastasis through modulation of the PI3K/AKT cascade in OSCC cells [29]. Laminin subunit β3 (LAMB3) promotes cell survival, invasion, and metastasis through the PI3K/Akt axis in patients with pancreatic cancer [30]. Procollagen-lysine,2-oxoglutarate 5-dioxygenase 1 can facilitate cell growth and aerobic glycolysis through activating the PI3K/Akt/mTOR signaling in gastric cancer cells [31]. Homer protein homolog 3 enhances EGF-induced aggressiveness and metastasis via activation of β-catenin in triple negative breast cancer cells [32]. In addition, using bioinformatics analysis, the genes co-expressed with MYO1B or pleckstrin-2 are highly associated with the activation of PI3K/AKT pathway in HNSCC tissues [33, 34]. Collectively, the observations suggest that proteome profiling of primary and PDX cancer tissues can reveal the interplay between the OSCC-related proteins, PI3K/AKT, and β-catenin pathways.

In our previous work, 12 OSCC PDX models were successfully generated using the tumor tissues form 49 patients with OSCC. Among the 49 patients, primary tumor tissues of 11 grafters and 23 non-grafters underwent RNA-seq analyses. Transcriptome analysis demonstrated that activation of hypoxia, PI3K/AKT, and EMT pathways in primary tumors correlates with their higher capacity for PDX establishment. Primary tumors with higher expression of matrix metalloproteinase 1 gene have a greater advantage in xenografts [10]. Additionally, RNA-seq analysis was performed on the primary tumors and their matched PDX P1 and P2 tumors. A total of 650 upregulated genes (defined as > 2-fold change and *p* < 0.05) were conserved in the primary tumor, P1, and P2 tissues compared with noncancerous tissues. Pathway enrichment analysis revealed that the 650 upregulated genes were associated with cancer progression-related pathways, including cell cycle, ECM-receptor interaction, and PI3K/AKT pathways [10]. Consistent with the present proteome analysis, ENAH is one of the 650 upregulated genes, and the OSCC progression-related proteins are likely associated with ECM-receptor interaction, actin cytoskeleton regulation, and PI3K/AKT signaling pathways (Fig. 2B, C).

Human ortholog of mammalian enabled (hMENA), also known as ENAH, is a member of the enabled/vasodilator-stimulated phosphoprotein (Ena/VASP) family. Proteins of this family possess an N-terminal Ena/VASP homology (EVH) 1 domain for interactions between proteins, a C-terminal EVH2 domain for actin binding, and a central proline-rich domain for recruiting profilin. The C-terminal coiled-coil of the EVH2 domain accounts for the homo- and heterotetramerization of Ena/VASP proteins [35, 36]. This configuration allows the Ena/VASP proteins to act as processive actin polymerases to stimulate actin polymerization, accelerate filament elongation by barbed end capping proteins, and play crucial roles in modulating cell motility and adhesion [37, 38]. In addition, Ena/VASP proteins also regulate the activity of actin regulators (formins and Arp2/3) and promote the formation and dynamics of filopodia by accelerating actin polymerization and protein–protein interactions [36–40]. ENAH participates in modulating invasion and metastasis of cancer cells and is found to be dysregulated in breast, gastric, lung, and pancreatic cancers [15, 41–43]. Moreover, ENAH has been revealed as a transcriptional target of the Wnt/β-catenin pathway in gastric, colorectal, breast, and liver cancer cell lines [15, 19, 44]. Overexpression of ENAH has been associated with tumor progression and poor survival in OSCC [45]. Long non-coding RNA ENAH‐202, a transcript of *ENAH* gene, facilitated the progress of EMT by regulating ZNF502/VIM axis in OSCC [46]. However, there is limited information regarding mechanism underlying ENAH dysregulation in OSCC.

**Fig. 8:**
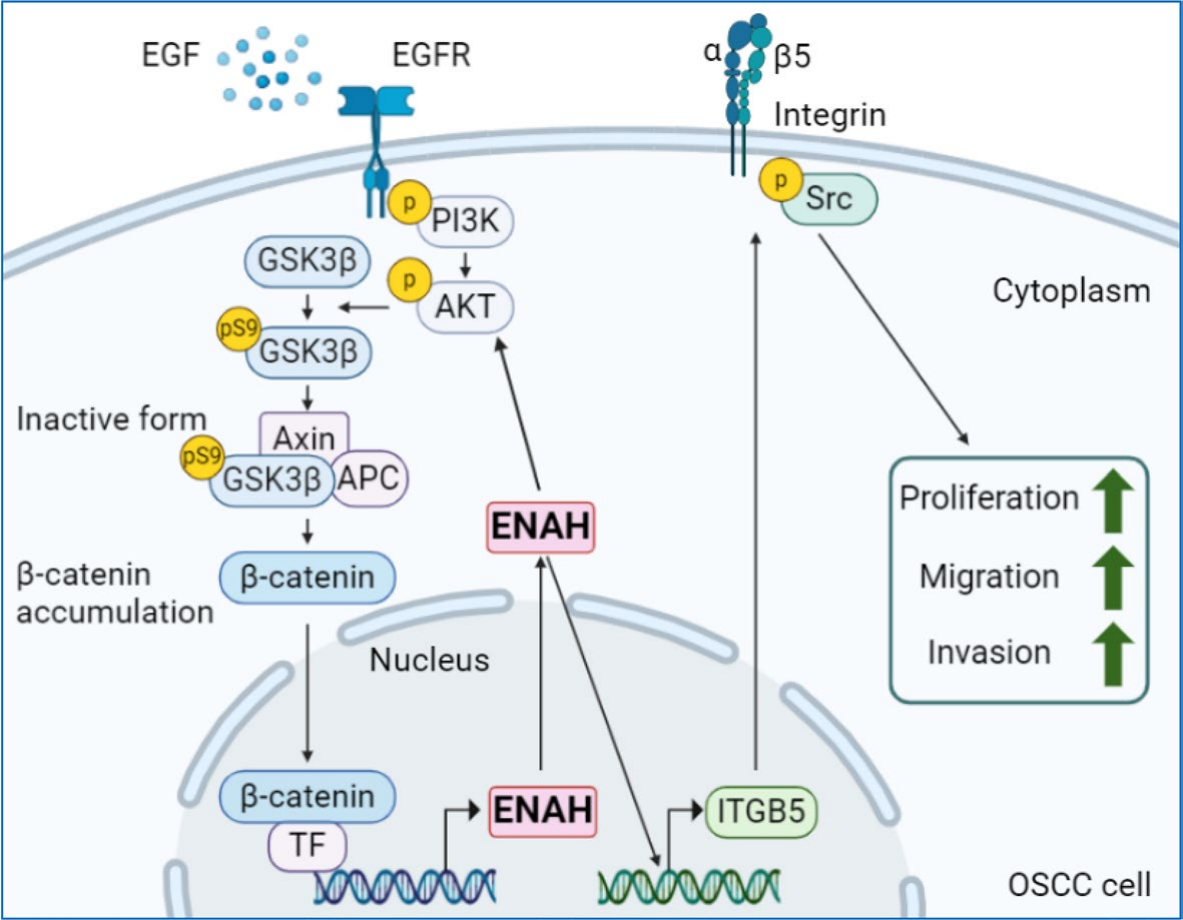
Schematic representation of ENAH-mediated OSCC progression. The EGFR signaling pathway plays a pivotal role in OSCC progression, inducing the activation of PI3K/AKT. Activated PI3K/AKT leads to the inactivation of GSK3β by phosphorylating its Ser 9. Inactivation of GSK3β results in β-catenin accumulation, which subsequently upregulates ENAH expression. Functionally, the elevated expression of ENAH enhances ITGB5 upregulation, leading to cell proliferation and migration, probably by modulating Src phosphorylation

Among proteins with increased levels in OSCC, ENAH has been selected for investigation due to its correlation with worse survival in gastric cancer patients [15], and its role in the actin-dependent cell migration [14]. Our findings suggest that ENAH expression is regulated by the EGFR/PI3K/AKT signaling pathway. Activation of PI3K/AKT leads to ENAH upregulation through the GSK3β/β-catenin axis. Previous studies have shown that β-catenin engages the DNA-bound transcription factor TCF in the nucleus [47], and ENAH has been identified as a transcriptional target of the Wnt/β-catenin/TCF4 complex in liver cancer cells [19], suggesting that ENAH is upregulated via β-catenin/TCF4 binding to the *ENAH* promoter in OSCC. Functionally, enhanced ENAH expression promotes ITGB5 upregulation and ITGB5-induced proliferation and motility in OSCC cells (Fig. 8). Our findings provide new insights into the role of ENAH in OSCC and uncover for the first time the mechanism driving ENAH-promoted OSCC progression.

As a protein associated with OSCC progression, ENAH is significantly increased in OSCC tissues and correlates with worse survival outcomes for cancer patients. Specifically, the ENAH level in OSCC tissue is a significant and independent predictor of adverse survival outcomes. However, the expression of ENAH in OSCC tissues does not show a significant correlation with well-established prognostic clinicopathological characteristics such as lymphatic metastasis, overall pathological stage, and perineural invasion. This discrepancy may be attributed to the differential expression of ENAH isoforms in OSCC tissues.

The ENAH gene undergoes alternative splicing to generate multiple protein isoforms (Supplementary Fig. S3A). For example, the hMENA^11a^ isoform, which includes exon 11a, has shown potential epithelial-specific, anti-apoptotic, and anti-migratory roles in cancers of the pancreas, lung, and breast [43, 48–50]. Conversely, the hMENAΔv6 isoform, which lacks exon 6, enhances the mesenchymal characteristics and invasive potential in cancer cells [43, 49, 51]. Four ENAH isoforms were detected in OSCC cell lines by immunoblotting (Supplementary Fig. S3B) and RT-PCR (data not shown). In addition to hMENA^11a^ and hMENA∆v6, a novel isoform named hMENA∆v6^11a^, which lacks exon 6 and contains exon 11a, was also found in OSCC cell lines. IHC analysis confirmed ENAH upregulation in OSCC tissues (Fig. 3C, D). Nevertheless, the antibody used for IHC analysis could not differentiate among the four ENAH protein isoforms. Utilizing antibodies that specifically recognize the various ENAH isoforms could establish a clearer link between the level of each ENAH isoform and OSCC prognostic factors. Additionally, ENAH isoforms are involved in processes that contribute to remodeling the tumor microenvironment (TME) by regulating the secretion of ECM components, including fibronectin and matrix metalloproteinase 2 [42]. However, the role of ENAH isoforms in shaping the OSCC TME remains poorly understood.

EGFR, a receptor tyrosine kinase, can turn on numerous signaling pathways, including the PI3K/AKT, MAPK, JAK/STAT and KRAS/BRAF/MEK/ERK cascades, and plays a role in cellular activities under physiological conditions. EGFR signaling is crucial in promoting the progression of various cancers, including OSCC, where it drives cell proliferation, apoptosis inhibition, chemoresistance, adhesion, and migration [18, 52, 53]. Therapeutic approaches, such as anti-EGFR antibodies and EGFR tyrosine kinase inhibitors, have been developed and have shown effectiveness in treating some patients with cancer. However, the therapeutic success of these treatments is limited in certain cases due to mutations and structural variants in the EGFR gene [54, 55]. Interestingly, our results indicate for the first time that ENAH expression enhances the phosphorylation of AKT, a downstream target of EGFR activation, in the OSCC cells (Supplementary Fig. S5). This suggests that the failure of EGFR-based cancer treatment may be partly attributed to ENAH-mediated AKT activation. Combining EGFR inhibition with targeting ENAH signaling could potentially improve the response rates to cancer treatments. Additional research is warranted to determine the involvement of ENAH in regulating EGFR-transduced signaling.

Integrins are a family of 24 heterodimeric transmembrane receptors composed of one α subunit and one β subunit. They are named based on the combination of their α and β subunits [21]. ITGB5 exclusively partners with ITGAV to form integrin αvβ5, which recognizes the extracellular matrix (ECM) via an Arg-Gly-Asp (RGD) motif [56]. IHC analysis using a specific antibody revealed that level of integrin αvβ5 in OSCC tissues was increased compared to noncancerous control tissues [57]. Consistent with these findings, we found that gene expression levels of *ITGAV* and *ITGB5* in OSCC tissues were higher than those in adjacent noncancerous tissues (Supplementary Table S8). Additionally, we discovered for the first time that ITGB5, but not ITGAV, was upregulated via ENAH expression in OSCC cells (Fig. 6 and Supplementary Fig. S6). These findings suggest that targeting integrin αvβ5 and ENAH downstream signaling could offer a viable approach to treating OSCC progression. Recently, cilengitide, a synthesized RGD-based peptide that selectively inhibits integrin αvβ5, has demonstrated significant antitumor activity in HNSCC cells [58]. Moreover, inhibitors targeting the EVH1 domain of Ena/VASP proteins have been developed and exhibited biological activity to suppress the migration of breast cancer cells [59]. However, research on the roles of these inhibitors in treating OSCC is still in the early stages and warrants further investigation.

## Conclusions

OSCC accounts for 2% of global cancer-related deaths each year. The poor prognosis of OSCC highlights the urgent need to decipher the mechanisms underlying its progression to develop effective therapeutic strategies. This work offers novel insights into the role of ENAH in OSCC progression. ENAH correlates with poorer survival rates in patients with OSCC. Upregulation of ENAH through a PI3K/AKT/β-catenin cascade enhances OSCC cell migration and growth via the ITGB5/Src axis (Fig. 8). Our findings deepen the understanding of ENAH’s role in OSCC progression, providing crucial information for developing treatment approaches.

## Supplementary Information

Supplementary material 1: Supplementary Materials and Methods

Supplementary material 2: Supplementary Figures

Supplementary material 3: Supplementary Table S1. Clinical characteristics of OSCC tissues used in iTRAQ analysis. Supplementary Table S2. List of qRT-PCR primers. Supplementary Table S7. The clinicopathological characteristics related to ENAH expression in tissue samples from 304 patients with OSCC. Supplementary Table S8. List of gene expression correlation between ENAH and integrin subunits in OSCC tissues

Supplementary material 4: Supplementary Table S3-1. List of proteins identified in iTRAQ analysis of OSCC #06 group

Supplementary material 5: Supplementary Table S3-2. List of proteins identified in iTRAQ analysis of OSCC #29 group

Supplementary material 6: Supplementary Table S3-3. List of proteins identified in iTRAQ analysis of OSCC #34 group

Supplementary material 7: Supplementary Table S3-4. List of proteins identified in iTRAQ analysis of OSCC #44 group

Supplementary material 8: Supplementary Table S3-5. List of proteins identifed in iTRAQ analysis of OSCC #48 group

Supplementary material 9: Supplementary Table S4. List of proteins quantified in iTRAQ analysis of OSCC #06, 29, 34, 44, and 48 groups

Supplementary material 10: Supplementaty Table S5. List of proteins differentially expressed in OSCC tissues

Supplementary material 11: Supplementary Table S6. Enrichment analysis of biological processes and biological pathways related to differentially expressed proteins in OSCC tissues

Supplementary material 12: Supplementary Material: Original immunoblots in figures

## Data Availability

All data generated in this study are included in this article and its supplementary files. Original immunoblots are provided in the supplementary information file. The raw data for the iTRAQ-based MS analysis have been deposited on the ProteomeXchange Consortium website under the PRIDE partner repository (data set identifier: PXD046930; http://proteomecentral.proteomexchange.org).

## Abbreviations

AKT: Protein kinase B
EGFR: Epidermal growth factor receptor
Ena/VASP: Enabled/vasodilator-stimulated phosphoprotein
ENAH: Protein enabled homolog
EVH: Ena/VASP homology
GSK3β: Glycogen synthase kinase 3β
HCL: Hierarchical clustering
hMENA: Human ortholog of mammalian enabled
HNSCC: Head and neck squamous cell carcinoma
IGF2BP2: Insulin-like growth factor 2 mRNA-binding protein 2
IHC: Immunohistochemistry
ITGB5: Integrin β5
iTRAQ: Isobaric tags for relative and absolute quantitation
KEGG: Kyoto Encyclopedia of Genes and Genomes
LC: Liquid chromatography
MYO1B: Myosin 1B
MS: Mass spectrometry
OSCC: Oral cavity squamous cell carcinoma
PCA: Principal component analysis
PDX: Patient-derived xenograft
PPI: Protein–protein interaction
TME: Tumor microenvironment
