# Supplementary material for: Upregulation of ENAH by a PI3K/AKT/β-catenin cascade promotes oral cancer cell migration and growth via an ITGB5/Src axis
Source: Cell Mol Biol Lett. 2024 Nov 7;29:136. doi: 10.1186/s11658-024-00651-0 (PMC11545229; doi:10.1186/s11658-024-00651-0)
Supplement: Supplementary file 1 — Supplementary material 1: Supplementary Materials and Methods [file 11658_2024_651_MOESM1_ESM.docx]

**Supplemental Materials and Methods**

**PDX model establishment**

NOD.Cg-*Prkdc*^scid^ *Il2rg^tm1Wjl^*/SzJ (NSG) mice were obtained from The Jackson Laboratory and housed in a specific-pathogen-free animal room (Laboratory Animal Center, Chang Gung University, Taoyuan, Taiwan). All animal experiments were approved by the Institutional Animal Care and Use Committee of Chang Gung University (Approval No: CGU106-114 and CGU107-074). For establishment of OSCC PDX, tumor tissues of OSCC patients were obtained, washed with PBS, and rinsed in PBS containing the antibiotic-antimycotic solution (Gibco, Waltham, MA, USA). The tumor tissues were then cut into small pieces (~1 mm^3^) and surgically transplanted into left flanks of NSG mice within 2 hours of tissue specimen collection. Each NSG mouse was subcutaneously inoculated with approximately 80 mg of tumor pieces to establish first-generation (P1) PDX. When the tumors reached an average volume of 1000 mm^3^ in the P1 PDX mice, the P1 tumors were collected, rinsed in the antibiotic-antimycotic solution, cut into small pieces (~1 mm^3^), and then transplanted into the left flanks of NSG mice to establish the second generation (P2) PDX mice. For proteome analysis, the tumors grown to around 500 mm^3^ in PDX mice were collected to perform iTRAQ-based mass spectrometry analysis. Tumor size of PDX mice was measured with calipers, and the volume was calculated as follows: volume = 1/2 × length × width^2^.

**Tryptic digestion of tissue proteins and iTRAQ labeling**

Tissue pieces (approximately 3×3×3 mm³) of cancerous and adjacent noncancerous counterparts were flash-frozen in liquid nitrogen and homogenized with 300 μL of RapiGest™ SF (0.1%, Waters Corporation, Milford, MA, USA) using a Precellys 24 tissue homogenizer (Bertin Technologies, Ozyme, France). After centrifugation at 14,000 rpm and 4°C for 10 minutes, the resulting supernatant was harvested and stored in a -80°C freezer until use.

For protein digestion with trypsin, 10 μg of protein from each tissue sample was reduced with 250 mM tris-(2-carboxyethyl)-phosphine hydrochloride (Sigma-Aldrich, St. Louis, MO, USA) in 300 mM triethylammonium bicarbonate (TEABC, Sigma-Aldrich) at 60°C for 60 minutes, then alkylated for 30 minutes at room temperature with 300 mM TEABC containing 200 mM S-methyl methanethiosulfonate (Sigma-Aldrich). Protein digestion was carried out with Sequencing Grade Modified Trypsin (Promega, Madison, WI, USA) at 37°C overnight.

To profile the tissue proteomes, tryptic peptides were labeled with iTRAQ reagents according to the manufacturer's instructions (AB Sciex, Foster City, CA, USA). Briefly, the iTRAQ reagents were reconstituted in ethanol. The iTRAQ 114, 115, 116, and 117 tags were mixed with the peptides from the N, T, P1, and P2 samples of each patient, respectively, and incubated at room temperature for 1 hour. The iTRAQ-labeled samples were then frozen, dried using a Proteomic CentriVap Concentrator System (Labconco, Kansas City, MO, USA), and reconstituted in 0.1% trifluoroacetic acid (TFA).

The iTRAQ-tagged samples from the same patients were combined and desalted using C18 Ziptips (GE Healthcare Life Sciences, UK). The peptides were eluted with 1% TFA containing 30%, 50%, and 70% acetonitrile (ACN). The eluates were dehydrated using the Proteomic CentriVap Concentrator System and stored at -20°C.

**Peptide fractionation and LC-MS/MS analysis**

Online two-dimensional liquid chromatography (LC) separation was conducted using an UltiMate™ 3000 RSLCnano System (Thermo Fisher Scientific, San Jose, CA, USA) as previously described [1]. The iTRAQ-labeled samples were dissolved in 50 μL of 30% acetonitrile (ACN) containing 0.1% formic acid (FA), then injected into a strong cation exchange (SCX) column (Luna 5 µm SCX, 150 × 4.6 mm; Phenomenex, Taipei, Taiwan) at a flow rate of 1 μL/min. The peptides were eluted with a 5-99% gradient of 30% ACN containing 0.1 M ammonium chloride and 0.1% FA. The collected peptide fractions were diluted inline before being loaded onto a trap column (Zorbax 300SB-C18, 0.3 × 5 mm; Agilent Technologies, Wilmington, DE, USA). The trapped peptides were then eluted into a homemade reverse-phase (RP) column (HydroRP 2.5 μm, internal diameter: 75 μm, length: 20 cm) equipped with a 15-μm tip using solvent A (ACN containing 0.1% FA). The RPLC gradient was programmed at a flow rate of 0.3 μL/min as follows: 0-5 min, 0-5% A; 5-35 min, 5-28% A; 35-45 min, 28-40% A; 45-50 min, 40-60% A; 50-53 min, 60-90% A; 53-55 min, 90-5% A; 55-65 min, 5% A.

All MS data were acquired using an LTQ-Orbitrap Elite mass spectrometer (Thermo Fisher Scientific) with the operational software Xcalibur (version 2.2 SP1.48, Thermo Fisher Scientific). Full-scan MS was conducted in the Orbitrap with a scan range of 350-2000 Da and a resolution of 60,000 at *m/z* 400. Three *m/z* values (445.120025, 462.146574, and 536.165365) of (Si(CH_3_)_2_O)_6_H^+^ were used as internal standards for a mass lock.

Tandem MS data were acquired in a data-dependent mode, where the six most abundant ions in the preview MS scan were selected for collision-induced dissociation (CID) and higher-energy CID (HCD). With a relative mass window of 1.5 Da, ions selected for tandem MS were dynamically excluded for 3 min. The electrospray voltage was set at 1.8 kV, and the capillary temperature was 220°C. To prevent ions from overfilling the ion trap, the maximum ion numbers/accumulation times were set at 2 × 10^6^ ions/1000 ms for MS scan, 5 × 10^3^ ions/150 ms for CID, and 3 × 10^4^ ions/300 ms for HCD in automatic gain control configuration.

**Protein database searching and quantification**

Protein identification and iTRAQ-based quantification were performed using Proteome Discoverer software (version 1.4.1.14; Thermo Fisher Scientific). MS/MS spectra were searched against the Swiss-Prot human protein database (released March 2023) using Mascot software (version 2.2.0; Matrix Science, London, UK). For peptide-spectra matches, mass tolerances were set to 10 ppm for intact peptides, 0.5 Da for CID ions, and 0.05 Da for HCD ions. Fixed modifications included the addition of a methylthio group to cysteine (+46.0916 Da) and an iTRAQ tag to the N-terminus or lysine (+144.1544 Da). The oxidation of methionine (+15.9994 Da) was considered a variable modification. One missed tryptic cleavage site was allowed.

Furthermore, the MS/MS spectra were searched against a decoy protein database to evaluate the false discovery rate (FDR). The FDR was calculated by comparing the number of proteins identified in the decoy database to those in the Swiss-Prot database. To ensure an overall FDR below 0.01, the peptide confidence criteria included a peptide length >7 amino acids, peptide confidence *p*-value < 0.01, and at least two peptide hits per protein. Keratins were excluded from the list of identified proteins.

ITRAQ data were extracted from Proteome Discoverer to Microsoft Excel for protein quantification. Proteins with at least 2 quantifiable spectra were considered quantifiable proteins. iTRAQ ratio of each protein was log2-transformed and normalized with the median log2 ratio of all proteins. The mean and standard deviation (SD) of all protein log2 ratios were calculated. Proteins with log2 ratios exceeding the mean ratio plus one standard deviation (SD) were considered overexpressed, while those with log2 ratios falling below the mean ratio minus one SD were regarded as underexpressed.

**Bioinformatics analysis**

Proteins quantified in at least 4 iTRAQ analyses of OSCC tissues were selected to perform hierarchical clustering (HCL) analysis and principal component analysis (PCA) using the Partek Genomics Suite (Partek Inc., St. Louis, MO, USA). Normalized log_2_ iTRAQ ratios were transformed to *Z* scores. Missing values were represented by assigning half the minimum iTRAQ ratios to facilitate visualization and comparison. For HCL analysis, unsupervised two-way clustering was performed using the Ward’s clustering method and Euclidean distance. Functional enrichment analysis of dysregulated proteins in OSCC tissues was conducted using GO term and biological pathway analyses through the STRING database (version 11.5; [https://version-11-5.string-db.org/](https://version-11-5.string-db.org/)). For data visualization and graphing, results of the enrichment analyses for the biological process and Kyoto Encyclopedia of Genes and Genomes (KEGG) pathway were processed using SRplot ([http://www.bioinformatics.com.cn/SRplot](http://www.bioinformatics.com.cn/SRplot)) [2]. The GEPIA2 website (http://gepia2.cancer-pku.cn/#index) was utilized to determine correlations between expressions of ENAH and integrin subunits in the TCGA HNSCC dataset.

**IHC** **and scoring of tissue ENAH**

IHC analysis was performed following previously established protocols [3]. Tissue ENAH expression was assessed using an antibody against ENAH (1:150 dilution; Cat. No. 26421-1-AP; Proteintech, Chicago, IL, USA). Expression of ENAH was assessed using a simplified H score system that takes into account both the staining intensity of ENAH and the percentage of ENAH-positive cells. Negative, weak, moderate, and strong staining of ENAH were assigned intensity scores of 0, +1, +2, and +3 respectively. For each intensity score, the percentage of ENAH-positive cells with the same staining intensity was estimated visually. For each case, the resulting ENAH IHC score was calculated as the sum of the percentage of stained cells multiplied by the intensity scores. For example, a case with 10%, 20%, 30%, and 40% of tumor cells stained negatively, weakly, moderately, and strongly, respectively, would be assigned an ENAH IHC score of 200 (10 × 0 + 20 × 1 + 30 × 2 + 40 × 3 = 200). The specimens were evaluated by Dr. K. P. Chang without prior knowledge of their clinical data.

For survival analysis, patient with an ENAH IHC score greater than 180 (the first tertile of IHC scores in 304 patients with OSCC) was considered to have high ENAH expression in cancer tissues, whereas scores below 180 were considered as low expression of ENAH.

**Western blot**

Cellular proteins separated by 10% SDS-PAGE were transferred onto PVDF membranes. The membranes were incubated with primary antibodies at 4°C overnight, followed by incubation with HRP-labeled secondary antibodies at room temperature for 1 hour. The protein of interest was visualized using an ECL substrate (Cat. No. WBKLS0500; Merck). The luminescence signal was detected using the Biostep Chemiluminescence Imager Celvin S 420 (Biostep, Burkhardtsdorf, Germany). The primary antibodies used included rabbit anti-AKT (1:3000; Cat. No. 9272; Cell Signaling, Danvers, MA, USA), rabbit anti-β-catenin (1:60000; Cat. No. 51067-2-AP; Proteintech), non-phospho (active) β-catenin (Ser33/37/Thr41) antibody (1:1000; Cat. No. 4270; Cell Signaling), rabbit anti-ENAH (1:8000; Cat. No. 26421-1-AP; Proteintech), mouse anti-GAPDH (1:3000; Cat. No. sc-32233; Santa Cruz Biotechnology, Santa Cruz, CA, USA), rabbit anti-GSK3β (1:1000; Cat. No. sc-9166; Santa Cruz Biotechnology), rabbit anti-ITGB5 (1:1000; Cat. No. 3629; Cell Signaling), rabbit anti-Src (1:3000; Cat. No. 2109; Cell Signaling), rabbit anti-VCP (1;8000; Cat. No. 10736-1-AP; Proteintech), phospho-Akt (Ser473) antibody (1:1000; Cat. No. 9271; Cell Signaling), phospho-GSK3α/β (Ser21/9) antibody (1:1000; Cat. No. 9331; Cell Signaling), phospho-GSK3α/β (Tyr279/Tyr216) antibody (Cat. No. 05-413; Merck), and phospho-Src (Tyr419) antibody (1:1000; Cat. No. 6943; Cell Signaling).

**Statistical analysis**

For proliferation assays, a representative experiment with four replicates per group is shown, and the results of two independent replicate experiments must show similar trends. For migration, invasion, and qRT-PCR assays, all comparisons were based on data from three independent experiments. Shapiro-Wilk test was performed to determine whether data are normally distributed with Prism software (version 9.0; GraphPad Software, La Jolla, CA, USA). A two-sided *p*-value smaller than 0.05 is deemed to be statistically significant. Results of Shapiro-Wilk test are listed below:

For proliferation assays, *p*-values of the siCtrl and siENAH groups are 0.7435 and 0.3299, respectively (**Fig. 4A**). *p*-values of the vector and pENAH groups are 0.5564 and 0.199, respectively (**Fig. 4D**). *p*-values of the vector and pITGB5 groups are 0.4027 and 0.4564, respectively (**Fig. 6D**). In **Fig. 7A**, *p*-values of the vector, pENAH, vector/GDC0941, and pENAH/GDC0941 groups are 0.369, 0.9999, 0.6499, and 0.6474, respectively. In **Fig. 7D**, *p*-values of the siCtrl/vector, siENAH/vector, siCtrl/pITGB5, and siENAH/pITGB5 groups are 0.0869, 0.5357, 0.5858, and 0.2701, respectively. In supplemental figures, *p*-values of the siCtrl and siENAH groups are 0.7338 and 0.516, respectively (**Supplemental Fig. S4A**). *p*-values of the vector and pENAH groups are 0.3114 and 0.7048, respectively (**Supplemental Fig. S4D**). *p*-values of the vector and pITGB5 groups are 0.2886 and 0.3163, respectively (**Supplemental Fig. S7C**).

For transwell migration assays, *p*-values of the siENAH (**Fig. 4B**), pENAH (**Fig. 4E**), and pITGB5 (**Fig. 6E**) groups are 0.2767, 0.3087, and 0.8923, respectively. In **Fig. 7B**, *p*-values of the pENAH/GDC0941, vector/GDC0941, and pENAH/GDC0941 groups are 0.2238, 0.0737, and 0.8706, respectively. In **Fig. 7E**, *p*-values of the siENAH/vector, siCtrl/pITGB5, and siENAH/pITGB5 groups are 0.7542, 0.9028, and 0.8868, respectively. In supplemental figures, *p*-values of the siENAH (**Supplemental Fig. S4B**), pENAH (**Supplemental Fig. S4E**), and pITGB5 (**Supplemental Fig. S6D**) groups are 0.8261, 0.8476, and 0.8319, respectively.

For transwell invasion assays, *p*-values of the siENAH (**Fig. 4C**), pENAH (**Fig. 4F**), and pITGB5 (**Fig. 6F**) groups are 0.1301, 0.2575, and 0.6851, respectively. In **Fig. 7C**, *p*-values of the pENAH/GDC0941, vector/GDC0941, and pENAH/GDC0941 groups are 0.3379, 0.9951, and 0.5835, respectively. In **Fig. 7F**, *p*-values of the siENAH/vector, siCtrl/pITGB5, and siENAH/pITGB5 groups are 0.3184, 0.2591, and 0.2722, respectively. In supplemental figures, *p*-values of the siENAH (**Supplemental Fig. S4C**), pENAH (**Supplemental Fig. S4F**), and pITGB5 (**Supplemental Fig. S6E**) groups are 0.5118, 0.306, and 0.0917, respectively.

For qRT-PCR analysis, *p*-values of the siCtrl and siENAH groups are 0.0547 and 0.41, respectively (left panel of **Fig. 6A**). *p*-values of the siCtrl and siENAH groups are 0.0961 and 0.2912, respectively (right panel of **Fig. 6A**). *p*-values of the vector and pENAH groups are 0.4219 and 0.0697, respectively (left panel of **Fig. 6B**). *p*-values of the vector and pENAH groups are 0.3854 and 0.3619, respectively (right panel of **Fig. 6B**). *p*-values of the siCtrl and siENAH groups are 0.8857 and 0.3187, respectively (left panel of **Supplemental Fig. S7A**). *p*-values of the siCtrl and siENAH groups are 0.1045 and 0.1909, respectively (right panel of **Supplemental Fig. S7A**). *p*-values of the vector and pENAH groups are 0.9312 and 0.2339, respectively (left panel of **Supplemental Fig. S7B**). *p*-values of the vector and pENAH groups are 0.122 and 0.8343, respectively (right panel of **Supplemental Fig. S7B**).

**References**

1. Chu HW, Chang KP, Hsu CW, Chang IY, Liu HP, Chen YT, et al. Identification of salivary biomarkers for oral cancer detection with untargeted and targeted quantitative proteomics approaches. Mol Cell Proteomics. 2019;18(9):1796-806.

2. Tang D, Chen M, Huang X, Zhang G, Zeng L, Zhang G, et al. SRplot: A free online platform for data visualization and graphing. PLoS One. 2023;18(11):e0294236.

3. Hsu CW, Chang KP, Huang Y, Liu HP, Hsueh PC, Gu PW, et al. Proteomic profiling of paired interstitial fluids reveals dysregulated pathways and salivary NID1 as a biomarker of oral cavity squamous cell carcinoma. Mol Cell Proteomics. 2019;18(10):1939-49.
