# Supplementary material for: Upregulation of ENAH by a PI3K/AKT/β-catenin cascade promotes oral cancer cell migration and growth via an ITGB5/Src axis
Source: Cell Mol Biol Lett. 2024 Nov 7;29:136. doi: 10.1186/s11658-024-00651-0 (PMC11545229; doi:10.1186/s11658-024-00651-0)
Supplement: Supplementary file 2 — Supplementary material 2: Supplementary Figures [file 11658_2024_651_MOESM2_ESM.pdf]

Supplemental Fig. S1

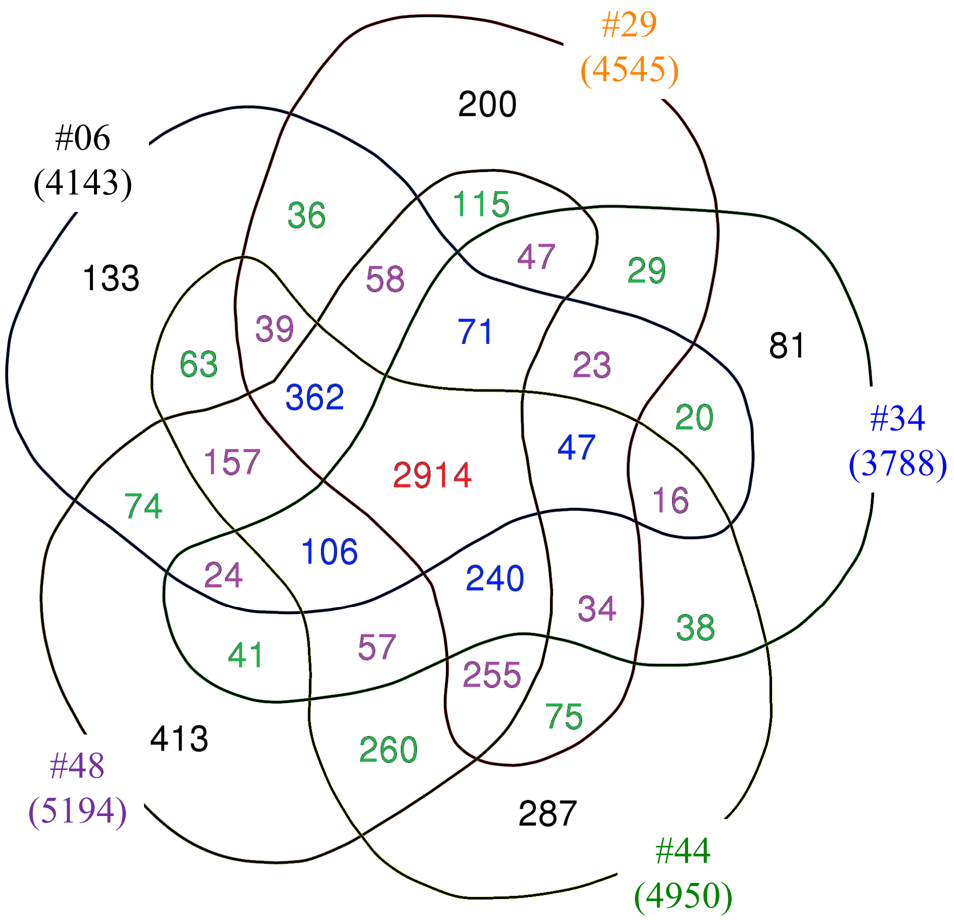

Supplemental Fig. S1. Proteome analysis of OSCC tissues with iTRAQ-based mass spectrometry. A Venn diagram illustrates number of protein quantified in 5 OSCC groups (#6, #29, #34, #44, and #48) with iTRAQ-based proteomics approach. Number in bracket represents number of proteins quantified in each OSCC group. Numbers marked in red, blue, purple, green, and black colors indicate proteins quantified in five, four, three, two, and one of five groups, respectively.

Supplemental Fig. S2

(A)

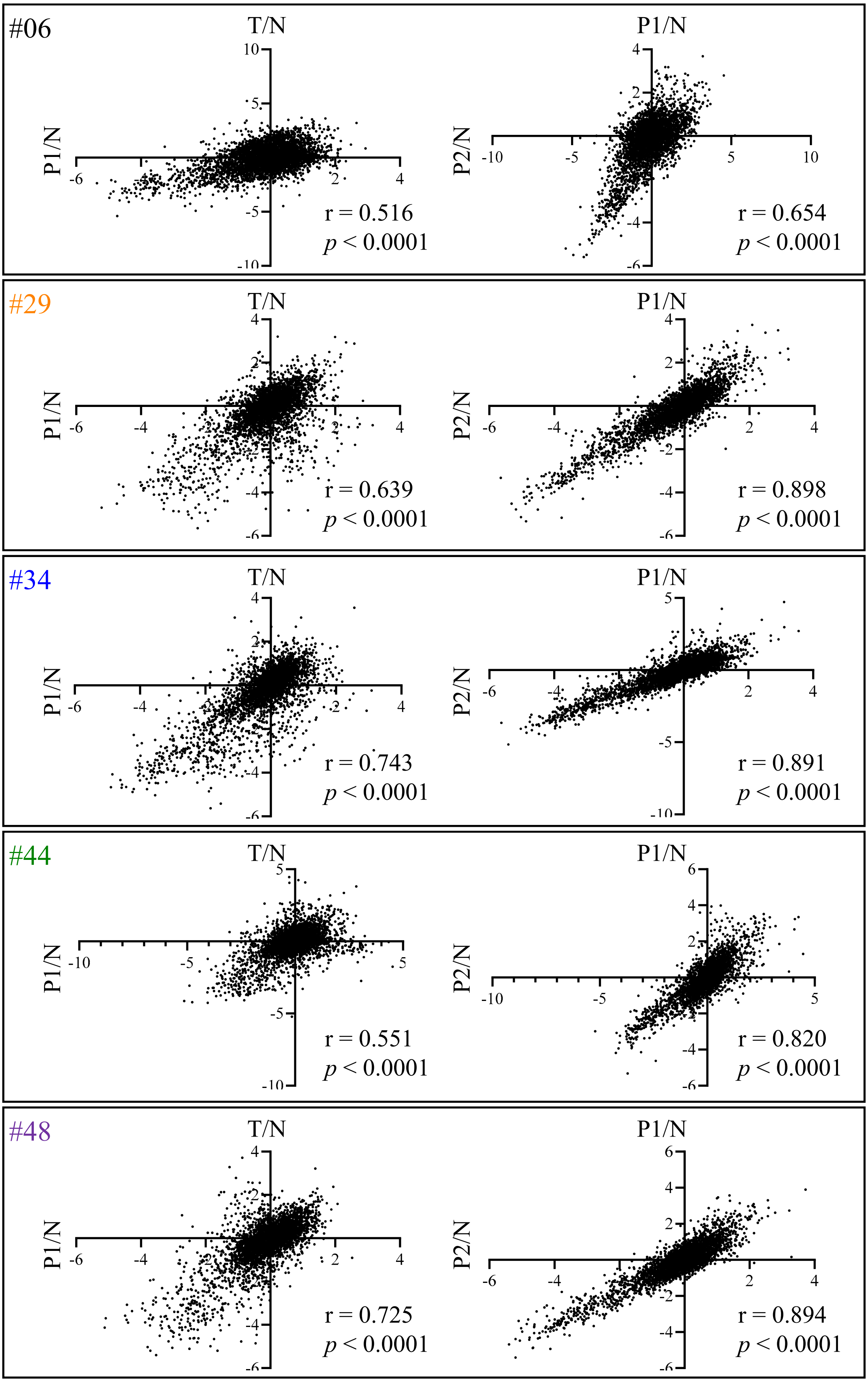

Supplemental Fig. S2 - continued

(B)

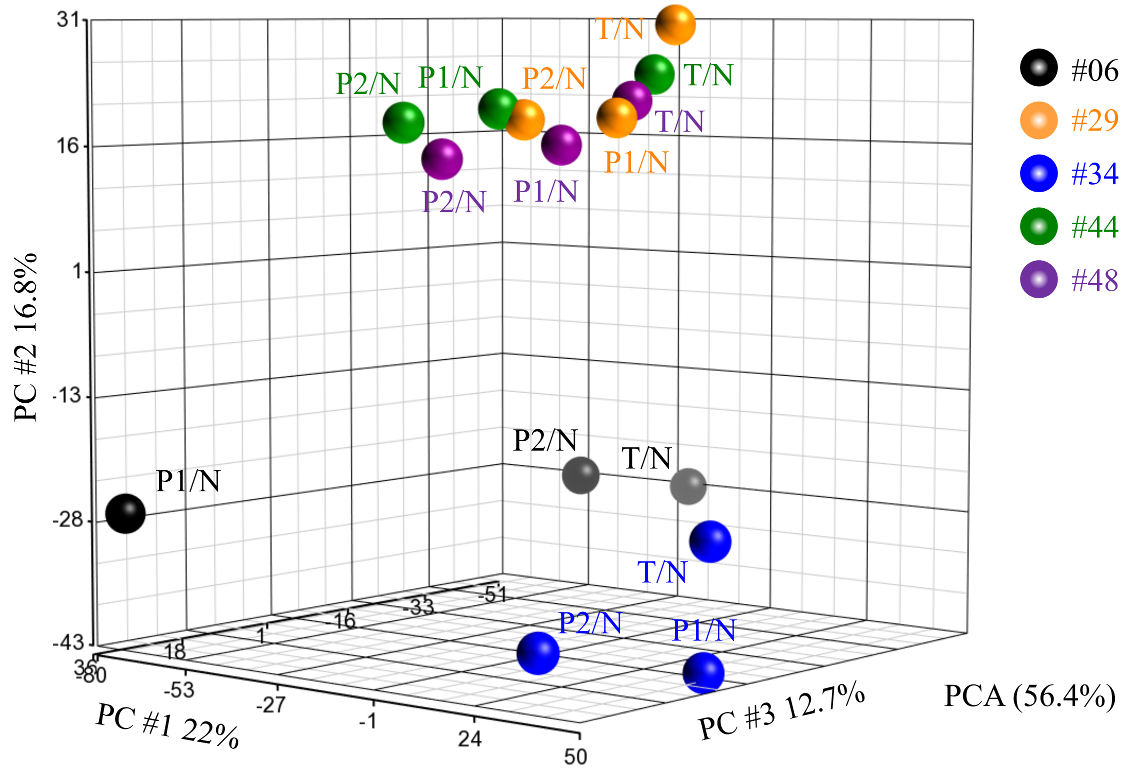

(C)

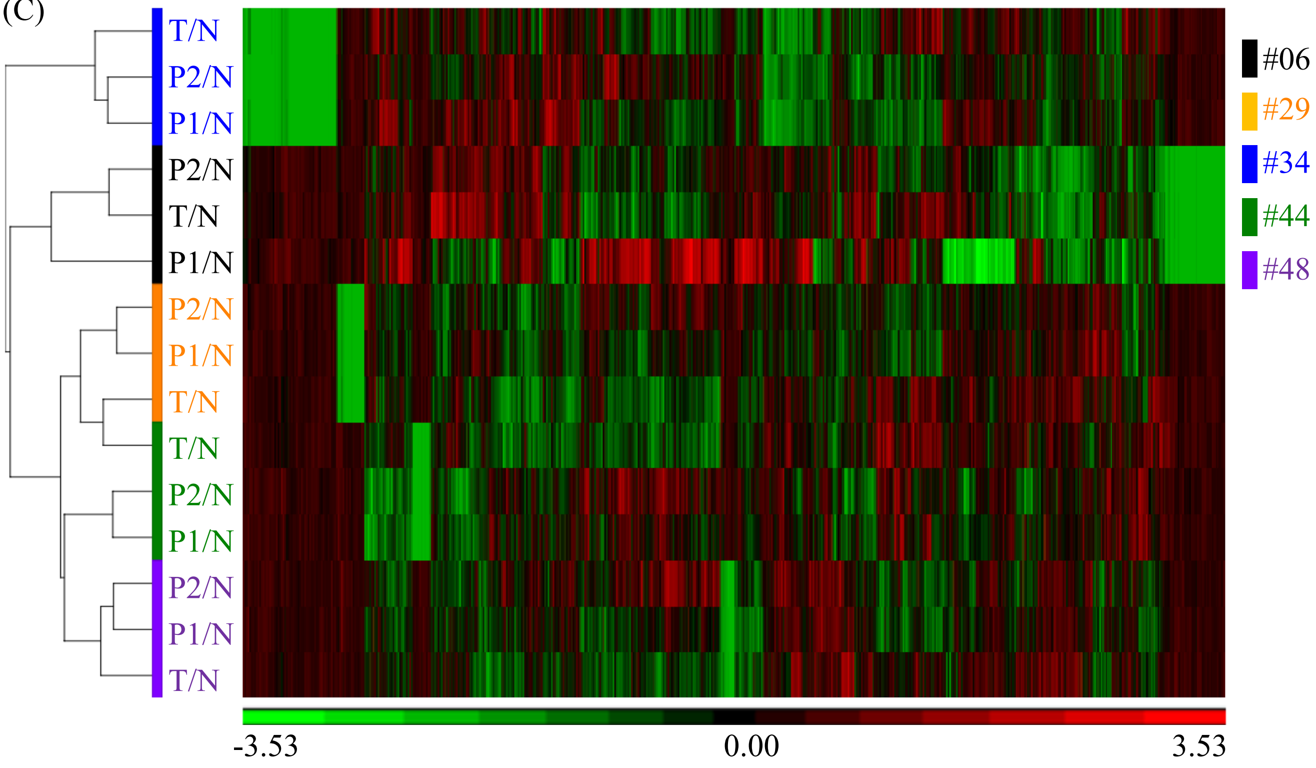

Supplemental Fig. S2. Quality assessment for proteome analyses of OSCC tissues. Proteome of tissues collected from 5 OSCC patients and their PDX models is profiled with iTRAQ-based mass spectrometry. (A) Correlations between protein ratios of T1/N and P1/N (left panel) and of P1/N and P2/N (right panel) are determined. (B, C) Principal component analysis (B) and hierarchical clustering analysis (C) are performed with the proteins quantified in at least 4 OSCC groups. N, non-cancerous tissue; T, primary tumor tissue; P1, tumor tissue of the 1st generation PDX; P2, tumor tissue of the 2nd generation PDX.

Supplemental Fig. S3

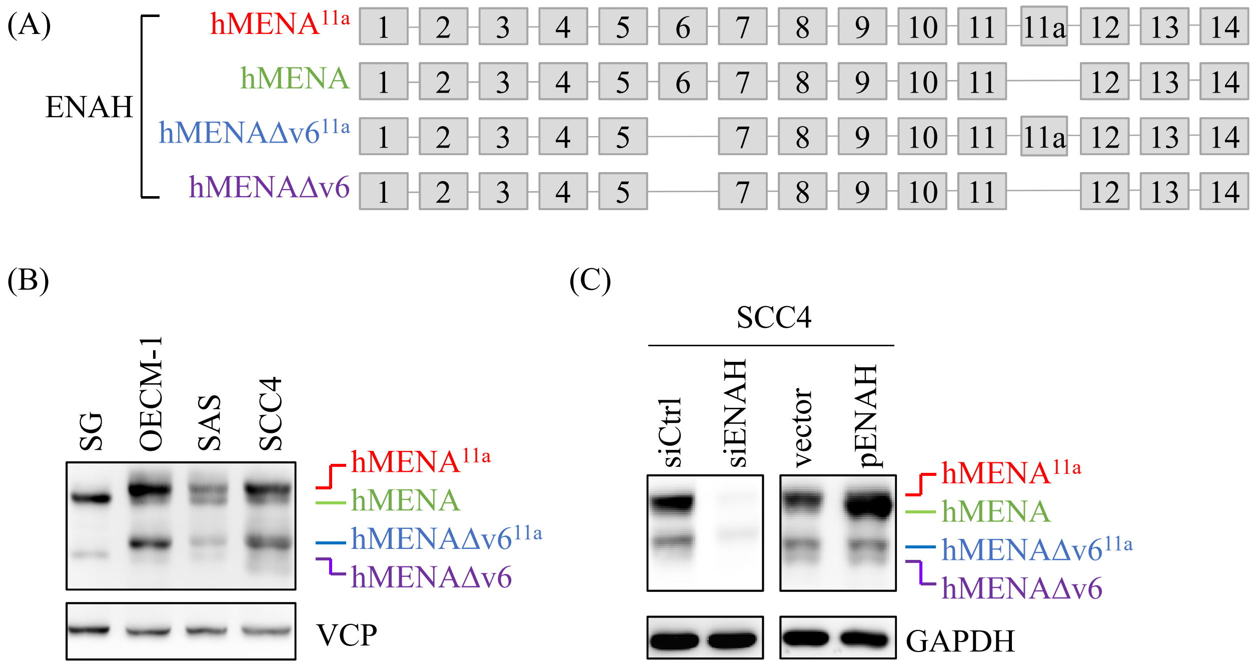

Supplemental Fig. S3. The isoforms of ENAH in OSCC cells. (A) Diagrammatic representation of 4 ENAH isoforms. (B) The 4 isoforms, hMENA, hMENA^{11a}, hMENAΔv6^{11a}, and hMENAΔv6, can be detected in 3 OSCC cell lines (OECM-1, SAS, and SCC4) and one non-cancerous oral epithelial cell line SG with immunoblotting. (C) The expression of 4 ENAH isoforms can be inhibited with the ENAH-specific siRNA in SCC4 cells. Coding sequences of hMENA was cloned in the pcDNA3.1C plasmid for ENAH overexpression in SCC4 cells.

Supplemental Fig. S4

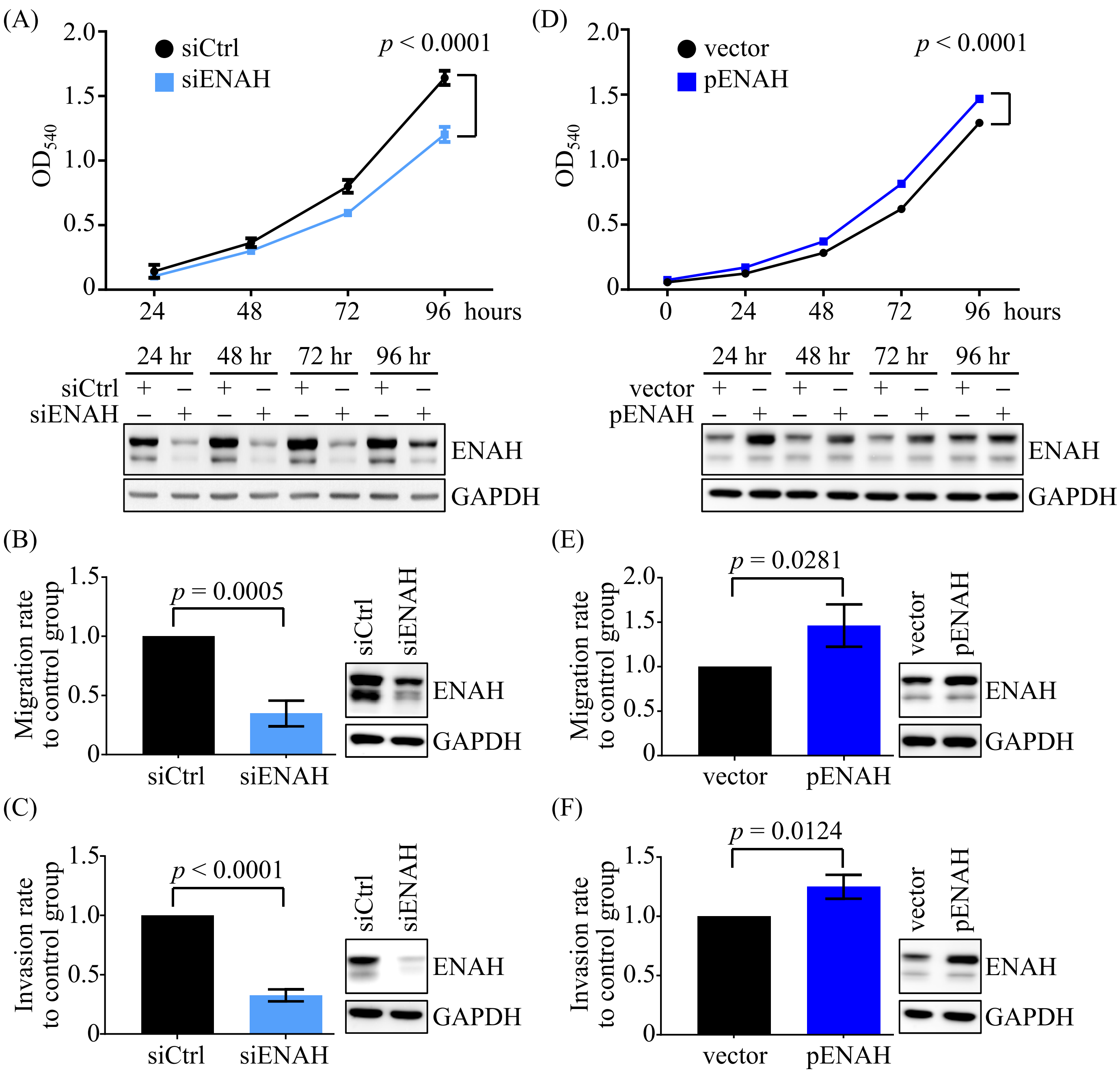

Supplemental Fig. S4. ENAH can enhance the ability of proliferation, migration, and invasion in OECM-1 cells. (A-C) The MTT cell proliferation (A), transwell migration (B), and matrigel invasion (C) assays of the control (siCtrl) and ENAH-knockdown (siENAH) OECM-1 cells were performed. (D-F) The abilities of cell growth (D), migration (E), and invasion (F) of the OECM-1 cells transfected with the control vector (vector) and ENAH expression plasmid (pENAH) were determined. Knockdown and overexpression efficacy was assessed by ENAH immunoblotting. For MTT assays, a representative experiment with four replicates per group is shown, and the results of two independent replicate experiments must show similar trends. For transwell assays, comparisons were based on data from three independent experiments. The *p*-values were determined using Student's *t*-test.

Supplemental Fig. S5

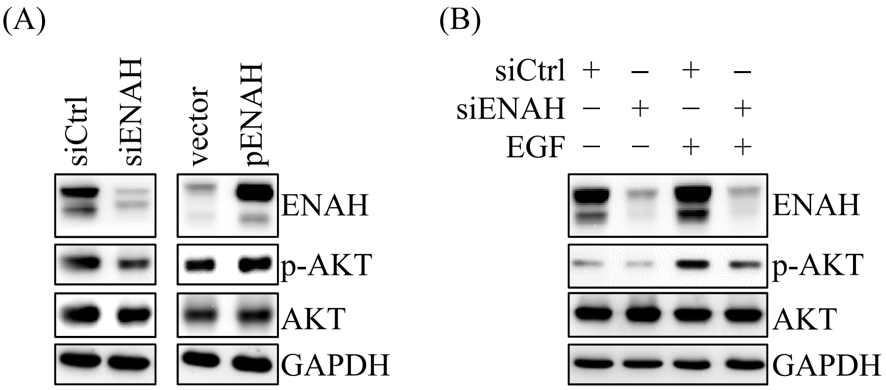

Supplemental Fig. S5. ENAH is involved in regulation of AKT phosphorylation. (A) To determine effect of ENAH expression on AKT phosphorylation, SCC4 cells were transfected with ENAH-specific siRNA (siENAH), control siRNA (siCtrl), ENAH-expressing plasmid (pENAH), and control vector (vector), respectively. The levels of p-AKT (Ser473), AKT, ENAH, and GAPDH were detected with the indicated antibodies. (B) AKT phosphorylation was reduced in ENAH-knockdown SCC4 cells with or without EGF (40 ng/mL) treatment.

Supplemental Fig. S6

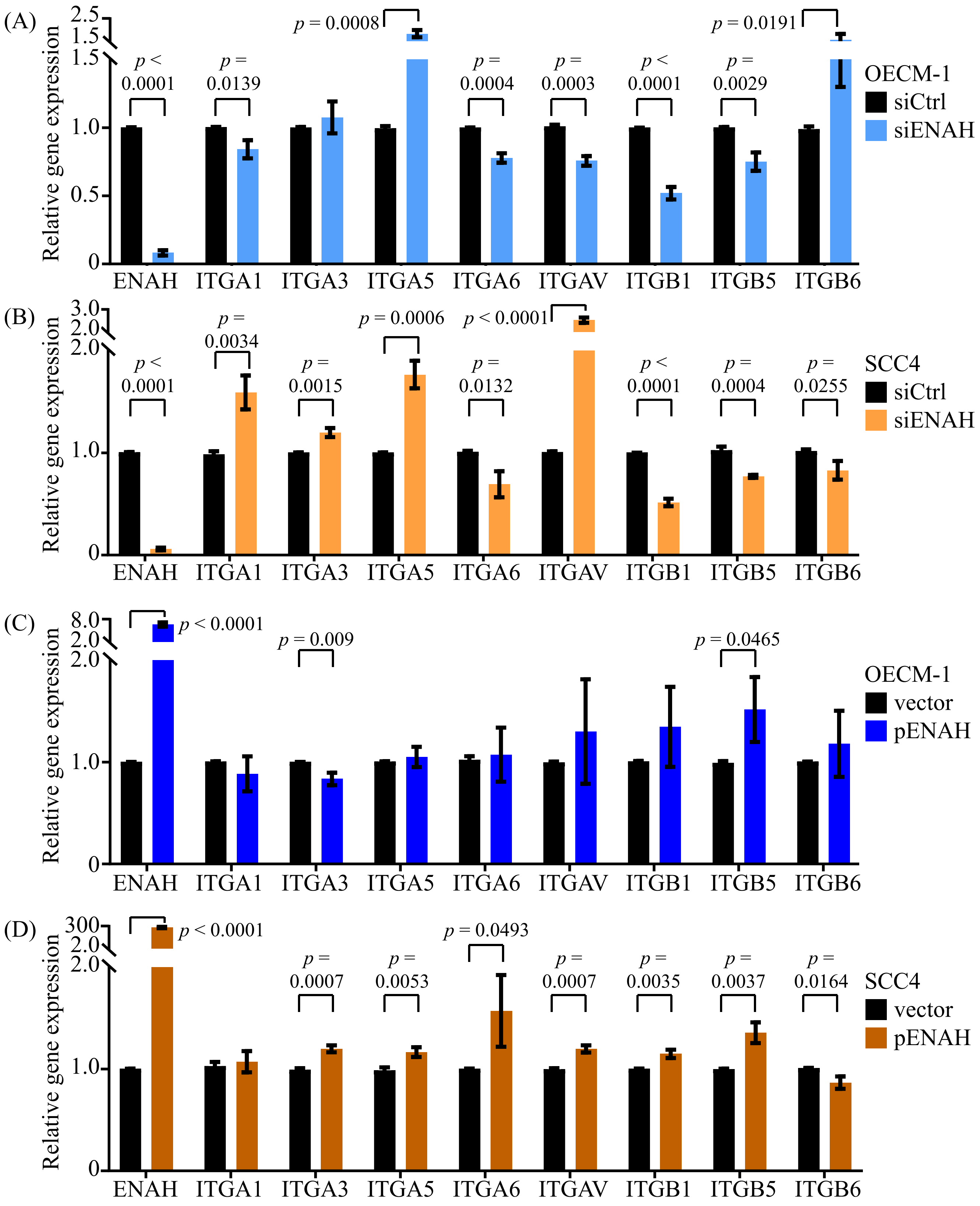

Supplemental Fig. S6. The effect of ENAH expression on mRNA levels of integrin subunits in OSCC cells. The gene expression of ENAH and integrin subunits is determined using qRT-PCR. ENAH expression is suppressed (A, B) and increased (C, D) in OECM-1 (A, C) and SCC4 (B, D) cells. The *p*-values of three independent experiments were determined by Student's *t* test.

Supplemental Fig. S7

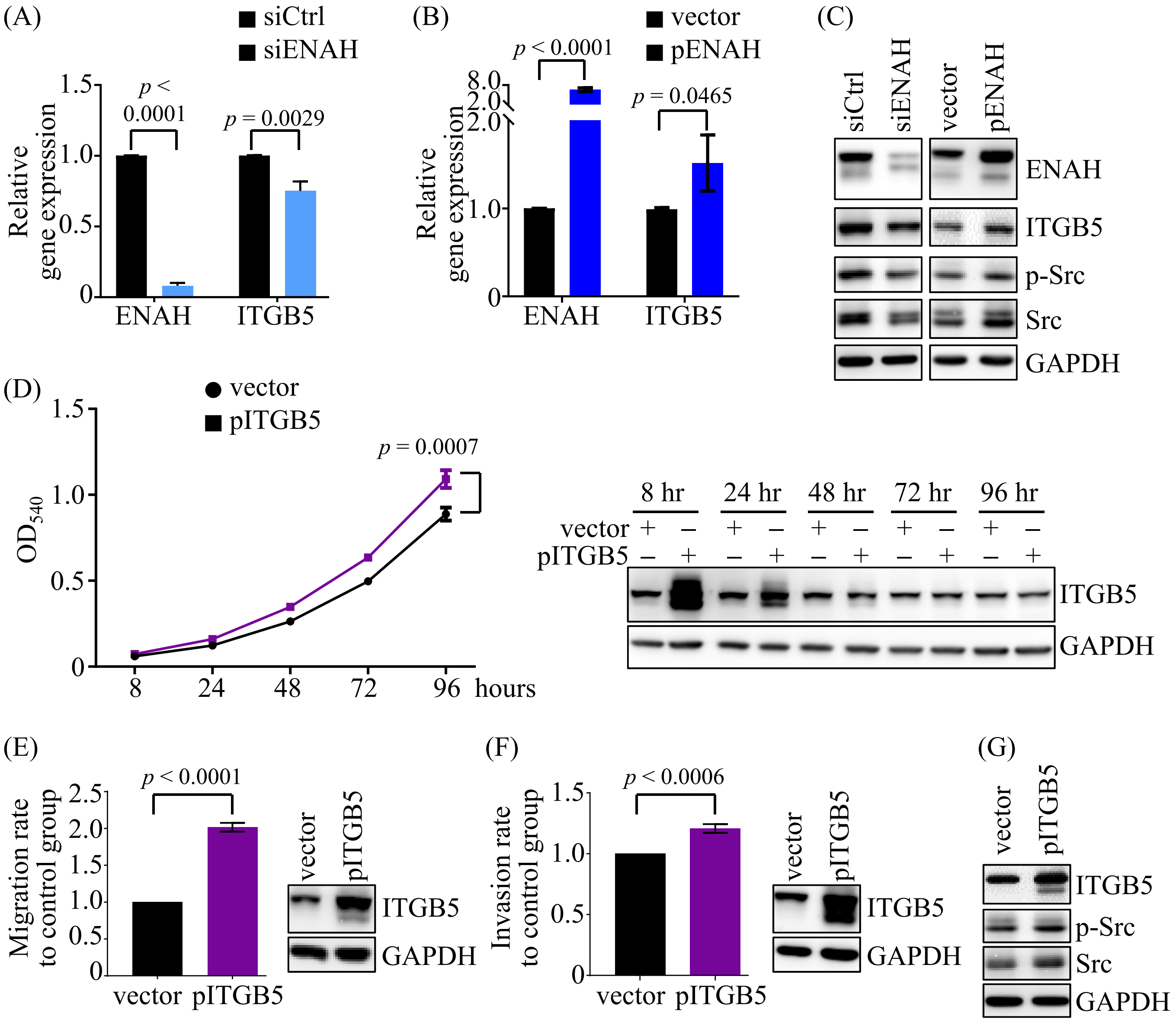

Supplemental Fig. S7. ENAH is involved in regulation of ITGB5 expression in OECM-1 cells. (A, B) The expression of ENAH and ITGB5 was detected in the OECM-1 cells transfected with the control siRNA (siCtrl), ENAH siRNA (siENAH), control vector (vector), and ENAH-expression plasmid (pENAH) using the qRT-PCR. (C) The levels of ITGB5 and p-Src (Tyr416) were verified in the ENAH-knockdown and ENAH-overexpressing OECM-1 cells. (D-F) The abilities of cell growth (D), migration (E), and invasion (F) of the OECM-1 cells transfected with the control vector (vector) and ITGB5-expression plasmid (pITGB5) were examined. (G) The levels of p-Src were investigated in the ITGB5-overexpressing OECM-1 cells. For MTT assays, a representative experiment with four replicates per group is shown, and the results of two independent replicate experiments must show similar trends. For qRT-PCR and transwell assays, comparisons were based on data from three independent experiments. The *p*-values of were determined using Student's *t*-test.
