# Supplementary material for: Upregulation of ENAH by a PI3K/AKT/β-catenin cascade promotes oral cancer cell migration and growth via an ITGB5/Src axis
Source: Cell Mol Biol Lett. 2024 Nov 7;29:136. doi: 10.1186/s11658-024-00651-0 (PMC11545229; doi:10.1186/s11658-024-00651-0)
Supplement: Supplementary file 3 — Supplementary material 3: Supplementary Table S1. Clinical characteristics of OSCC tissues used in iTRAQ analysis. Supplementary Table S2. List of qRT-PCR primers. Supplementary Table S7. The clinicopathological characteristics related to ENAH expression in tissue samples from 304 patients with OSCC. Supplementary Table S8. List of gene expression correlation between ENAH and integrin subunits in OSCC tissues [file 11658_2024_651_MOESM3_ESM.pdf]

Supplemental Table S1. Clinical characteristics of OSCC tissues used in iTRAQ analysis

OSCC ID	Sex	Age (year)	TNM stage				Cell differentiation	Habitual behaviors		
			T	N	M	Stage		Betel nut	Smoking	Alcohol
#06	Male	54	2	0	0	2	well	No	No	Yes
#29	Male	66	4A	0	0	4A	moderately	Yes	Yes	Yes
#34	Male	47	4A	2B	0	4A	moderately	Yes	No	Yes
#44	Male	40	2	0	0	2	moderately	Yes	Yes	No
#48	Male	49	3	1	0	4	moderately	Yes	Yes	No

Supplemental Table S2. List of qRT-PCR primers

Gene	Forward primer (5' to 3')	Reverse primer (5' to 3')
ENAH	CAGGAAACAGGGCCAACAT	TTGCCGTTGCTGTTCTTGTAG
β-catenin	GGGTCCTCTGTGAACTTGCT	CTGCATATGTCGCCACACC
ITGA1	ATCCCTTCCTGAGAAGAGGAG	GAGGTTCTTCGCTGTCACTTG
ITGA3	CAACCAGGATGGATTTCAGGA	CCCAGCTTCTCTCCATGGATTA
ITGA5	TTCACCAAGACTGGCCGT	CAGGTTGATCAGGTACTCGGG
ITGA6	AACTGGAAAGGGATTGTTCGTG	AGGAACGAGACTTTCATCATGC
ITGAV	CACCAACTCCACATTGGTTACC	CCAAAACAGCCAGTAGCAACAA
ITGB1	ACTTCGGACTTCAGAATTGGAT	AAATGGGCTGGTGCAGTT
ITGB5	TTGTCAGTCTGGGATCAGCC	ACTTCAAAAGATGCCGTGTCC
ITGB6	TGATCACGTACAAGGTGGCTG	GGTATCACACCTTTCGCCAAC
RPN18S	GCTTAATTTGACTCAACACGGGA	AGCTATCAATCTGTCAATCCTGTC
GAPDH	TCATTTCCTGGTATGACAACGA	TCTCTCTTCCTCTTGTGCTC

Supplemental Table S7. The clinicopathological characteristics related to ENAH expression in tissue samples from 304 OSCC patients

Characteristics	Case number	IHC score	
		Mean ± SD	*p*-value
Age (years)			
≤ 51.25	152	124.9 ± 77.7	0.1397
> 51.25	152	138.1 ± 76.5	
Sex			
Male	272	130.8 ± 77.2	0.6558
Female	32	137.2 ± 78.7	
pT Status			
1-2	154	128.6 ± 80.2	0.5049
3-4	150	134.5 ± 74.2	
pN Status			
N = 0	196	127.4 ± 76.1	0.2662
N > 0	108	138.9 ± 79.1	
Overall pathological stage			
I-II	111	128.9 ± 79.6	0.6752
III-IV	193	133.0 ± 76.0	
Perineural invasion			
No	189	128.3 ± 76.6	0.3557
Yes	115	136.8 ± 78.3	
Cell differentiation			
Well & moderate differentiation	272	132.9 ± 76.8	0.2869
Poor differentiation	32	119.5 ± 80.7	
Lymphovascular invasion			
No	285	130.9 ± 77.2	0.6055
Yes	19	140.3 ± 79.5	
Tumor depth			
< 10 mm	156	128.0 ± 82.9	0.3156
≥ 10 mm	148	135.2 ± 70.8	

Supplemental Table S8. List of gene expression correlation between ENAH and integrin subunits in OSCC tissues

Gene name	T/N[a]	Expression correlation with ENAH[b]	
		Pearson correlation coefficient	*p*-value
		Alpha (α) subunit	
**ITGA1**	↑	0.32	< 0.0001
ITGA2	↑	0.29	< 0.0001
**ITGA3**	↑	0.32	< 0.0001
ITGA4	↑	0.20	< 0.0001
**ITGA5**	↑	0.36	< 0.0001
**ITGA6**	↑	0.45	< 0.0001
ITGA7	−	0.04	0.43
ITGA8	−	0.13	0.0033
ITGA9	↓	0.15	0.00086
ITGA10	↑	0.06	0.21
ITGA11	↑	0.22	< 0.0001
ITGAE	−	-0.32	< 0.0001
ITGAL	−	-0.05	0.24
ITGAM	−	-0.02	0.72
**ITGAV**	↑	0.49	< 0.0001
ITGAX	↑	0.02	0.71
ITGA2B	ND	-0.18	< 0.0001
ITGAD	ND	-0.07	0.13
		Beta (β) subunit	
**ITGB1**	↑	0.45	< 0.0001
ITGB2	−	-0.02	0.66
ITGB3	−	0.25	< 0.0001
ITGB4	↑	0.05	0.28
**ITGB5**	↑	0.37	< 0.0001
**ITGB6**	↑	0.35	< 0.0001
ITGB7	−	-0.19	< 0.0001
ITGB8	−	0.15	0.0004

[a] The data was derived from RNA-Seq analysis of paired tumor (T) and adjacent non-cancerous (N) tissues from 33 OSCC patients (Yang et al., Front Oncol. 2022, 12, 792297). ↑, upregulation in OSCC tissues (T/N > 1.5 and $p < 0.05$); ↓, downregulation in OSCC tissues (T/N < 0.66 and $p < 0.05$); −, not dysregulation ($p > 0.05$); ND, not detected.

[b] The expression correlations between ENAH and integrin subunits in cancer tissues are determined with the TCGA dataset of head and neck cancer tissues (n = 519) using the GEPIA2 website.
