# Supplementary material for: Upregulation of ENAH by a PI3K/AKT/β-catenin cascade promotes oral cancer cell migration and growth via an ITGB5/Src axis
Source: Cell Mol Biol Lett. 2024 Nov 7;29:136. doi: 10.1186/s11658-024-00651-0 (PMC11545229; doi:10.1186/s11658-024-00651-0)

Fig. 4A ENAH

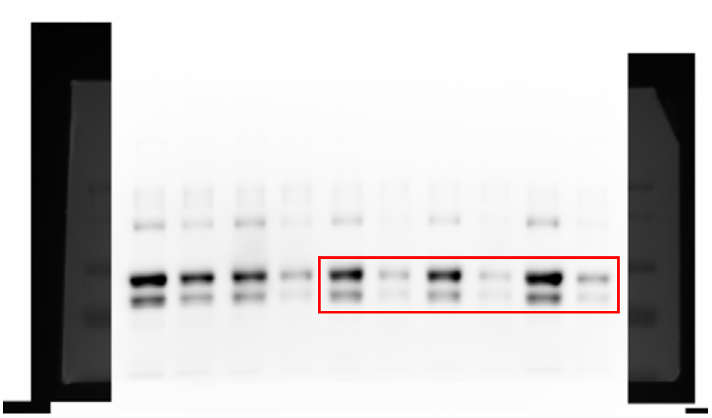

GAPDH

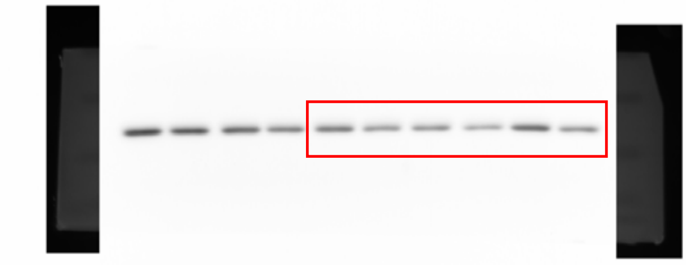

Fig. 4B ENAH

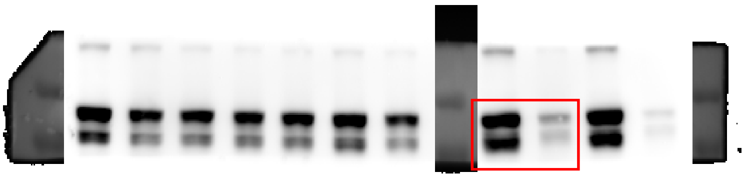

GAPDH

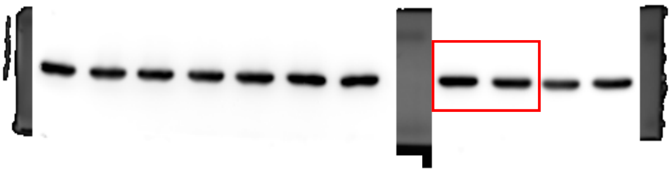

Fig. 4C ENAH

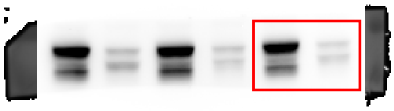

GAPDH

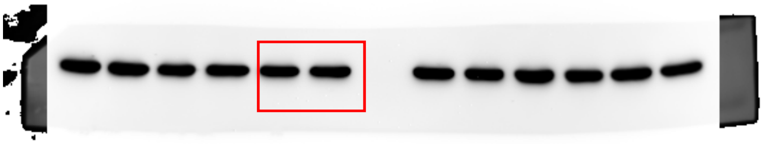

Fig. 4D ENAH

GAPDH

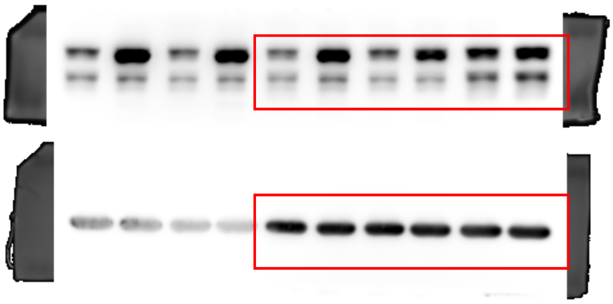

Fig. 4E ENAH

GAPDH

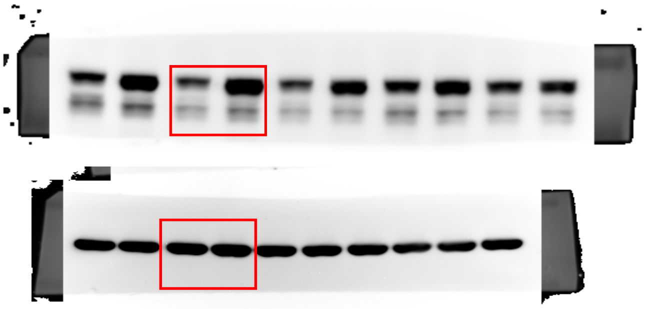

Fig. 4F ENAH

GAPDH

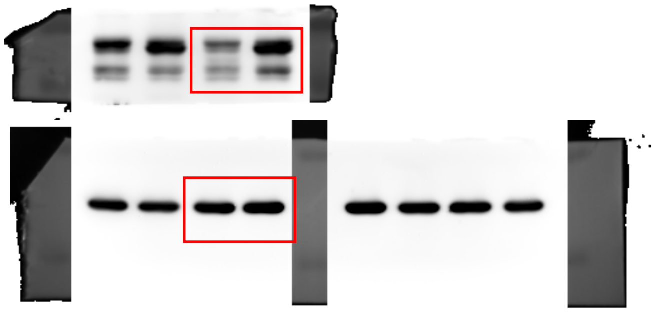

Fig. 5B

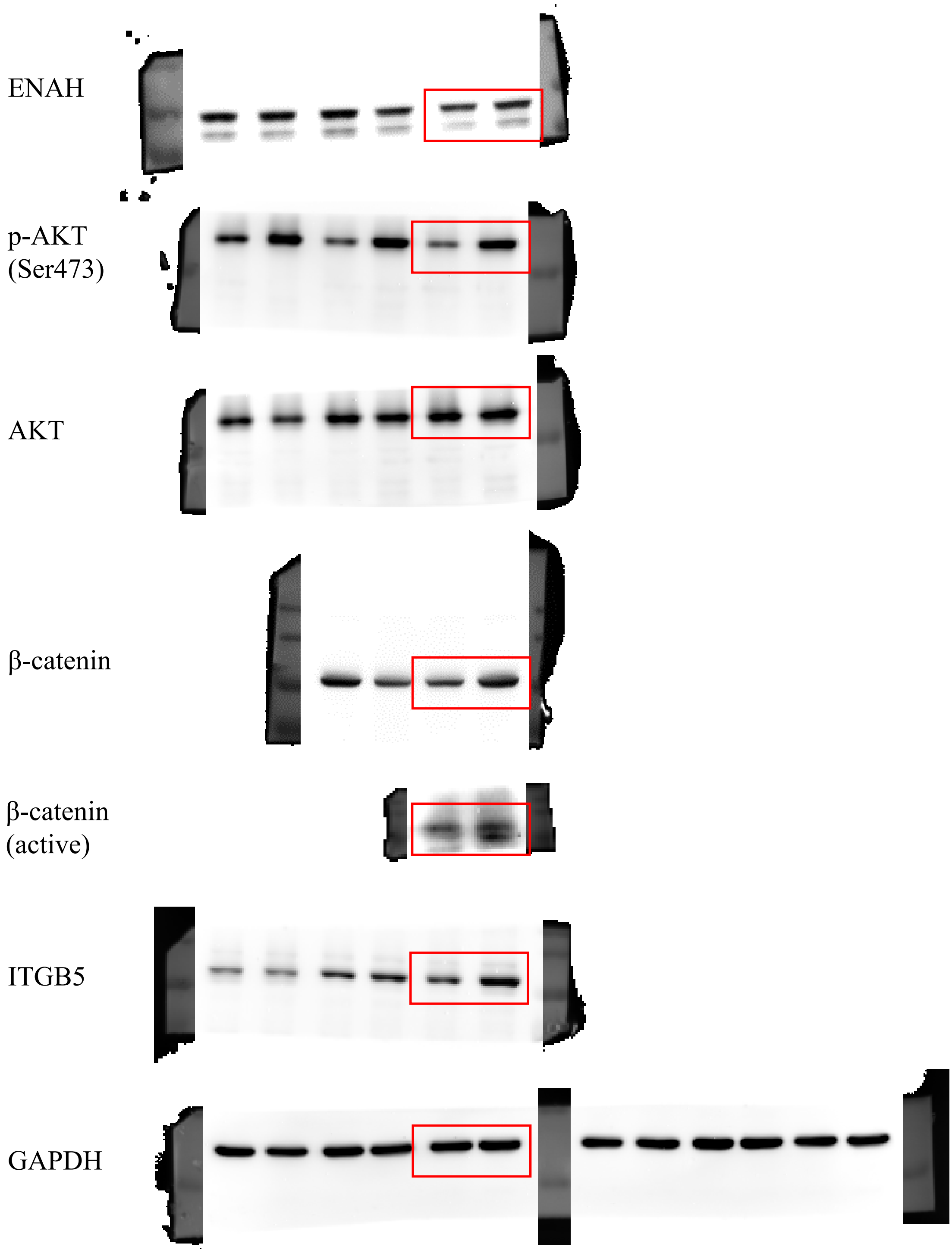

Fig. 5D

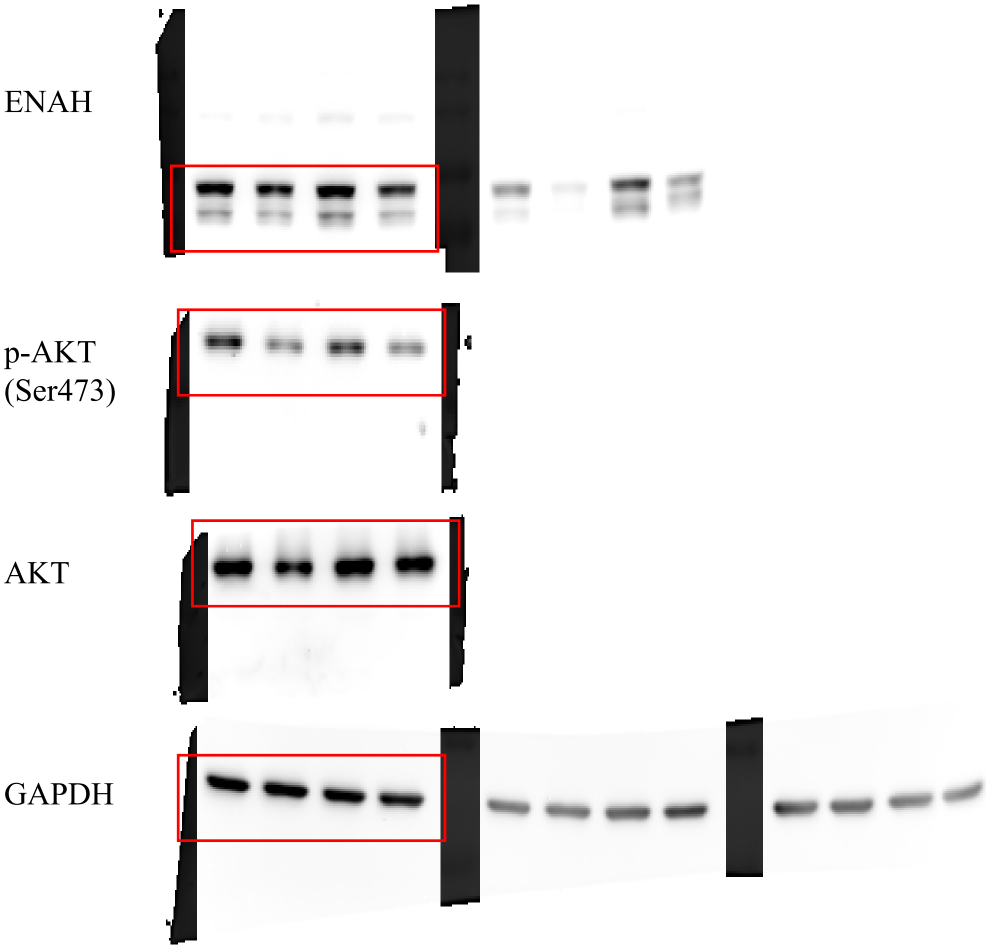

Fig. 5F

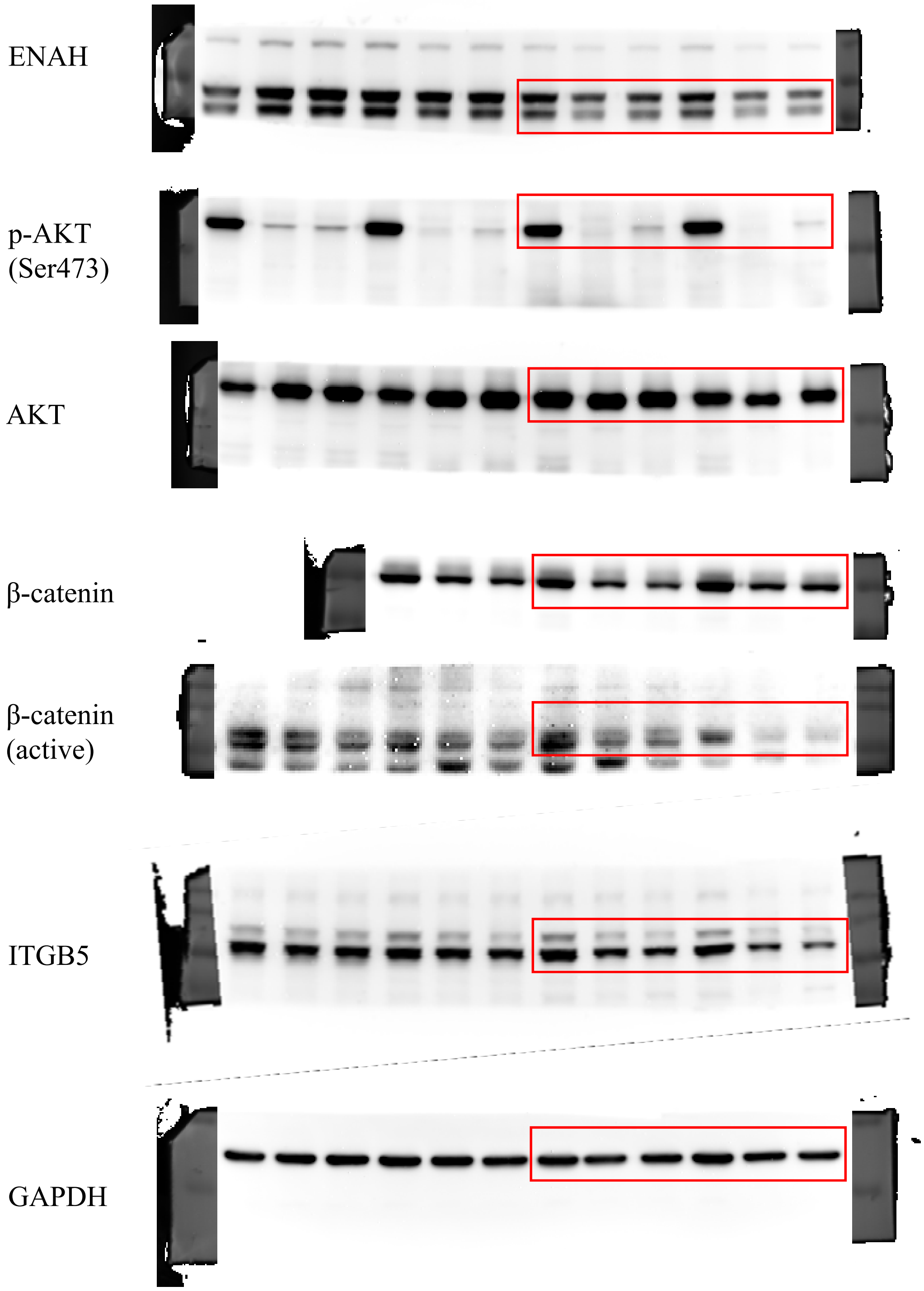

Fig. 5H ENAH

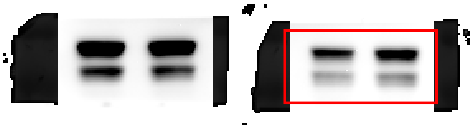

β-catenin

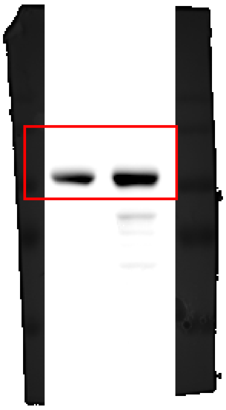

GAPDH

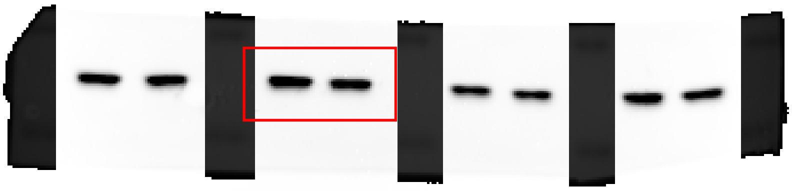

Fig. 5I ENAH

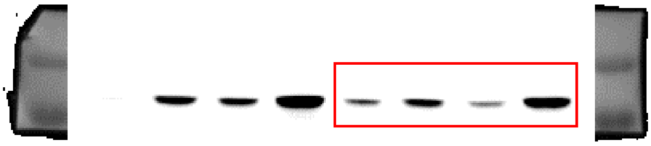

p-AKT (Ser473)

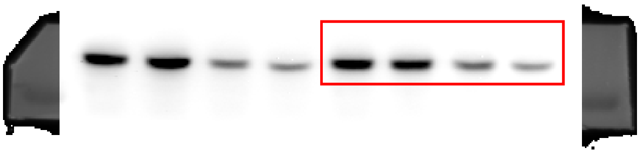

AKT

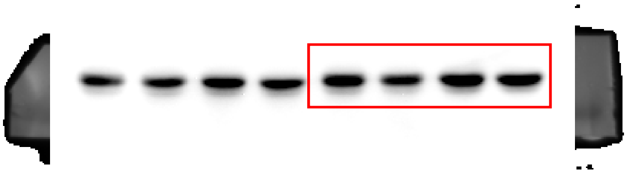

β-catenin

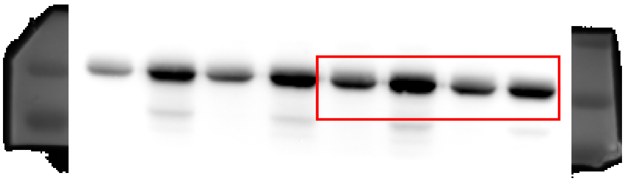

GAPDH

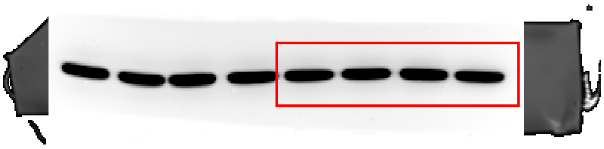

Fig. 5J

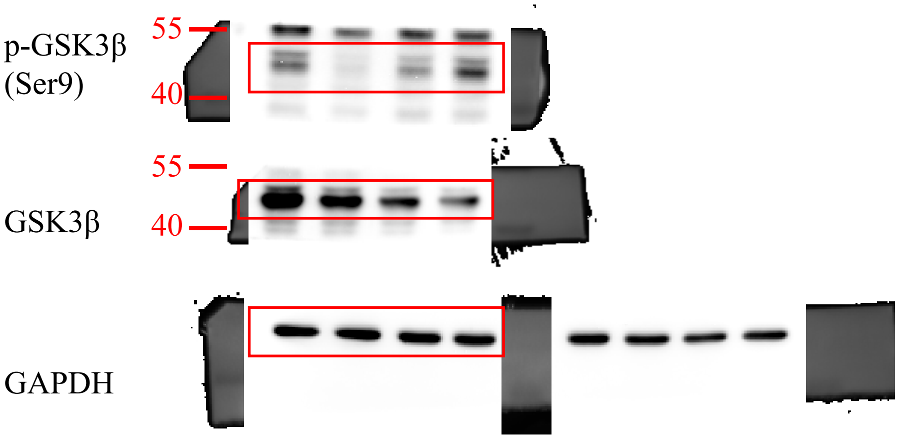

Fig. 6C (left Panel)

ENAH

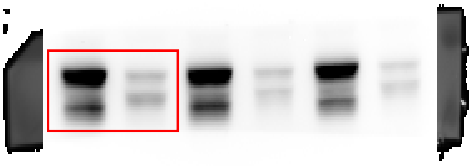

ITGB5

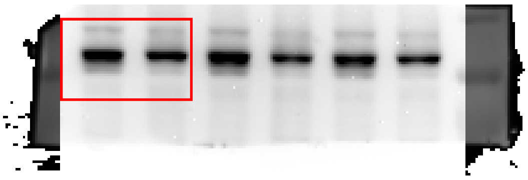

p-Src (Tyr419)

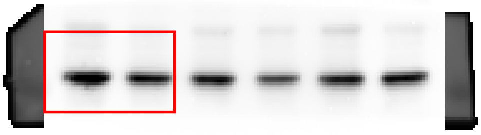

Src

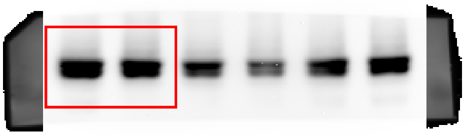

GAPDH

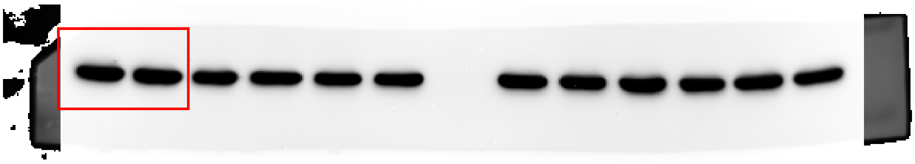

Fig. 6C (right Panel)

ENAH

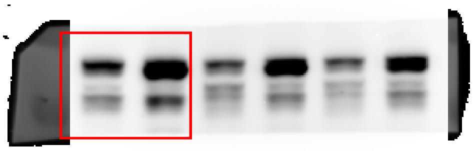

ITGB5

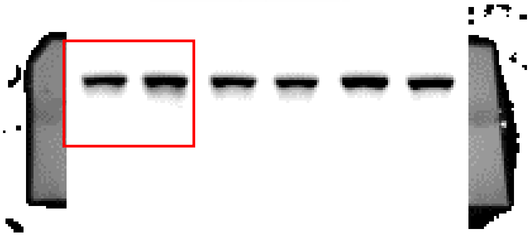

p-Src (Tyr419)

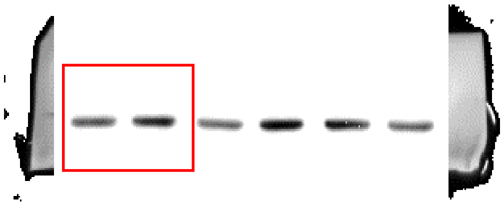

Src

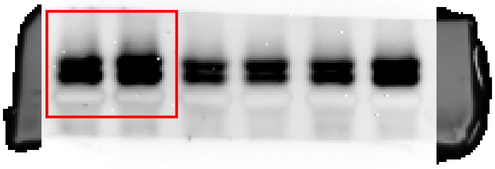

GAPDH

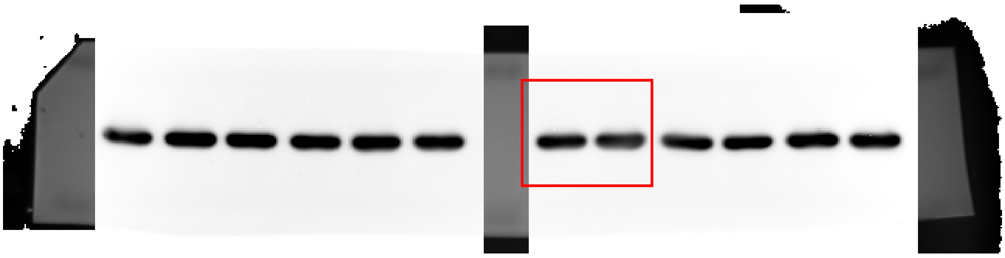

Fig. 6D

ENAH

p-AKT (Ser473)

AKT

ITGB5

GAPDH

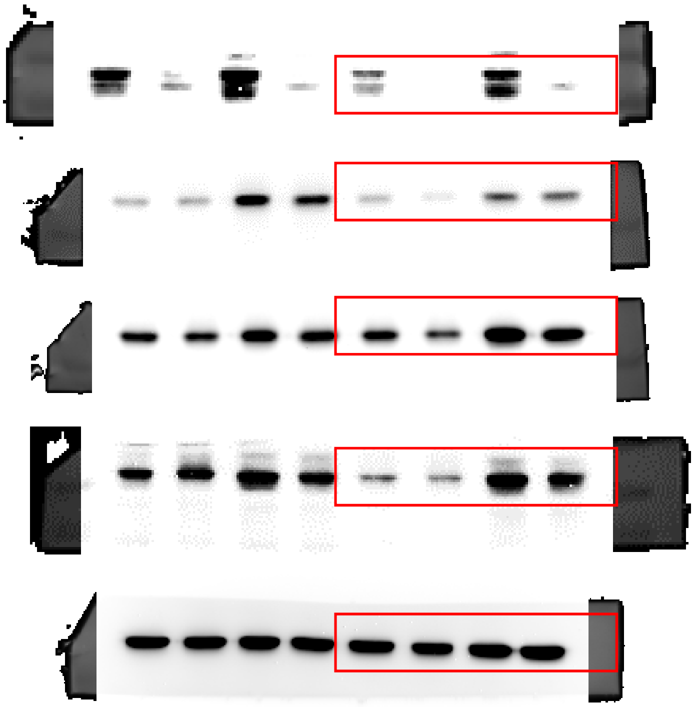

Fig. 6E

ITGB5

GAPDH

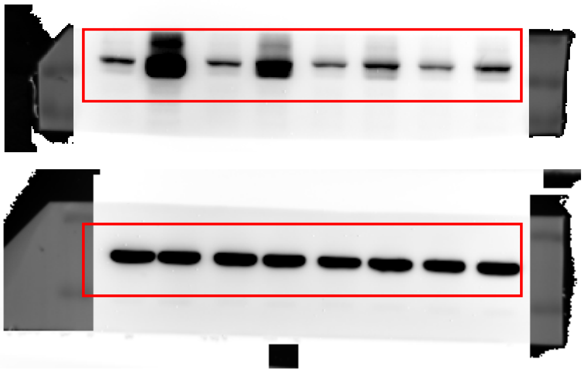

Fig. 6F

ITGB5

GAPDH

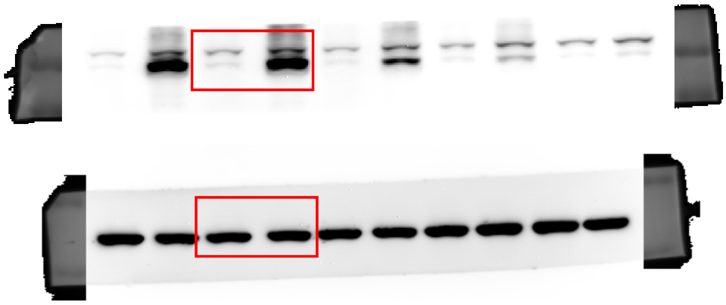

Fig. 6G

ITGB5

GAPDH

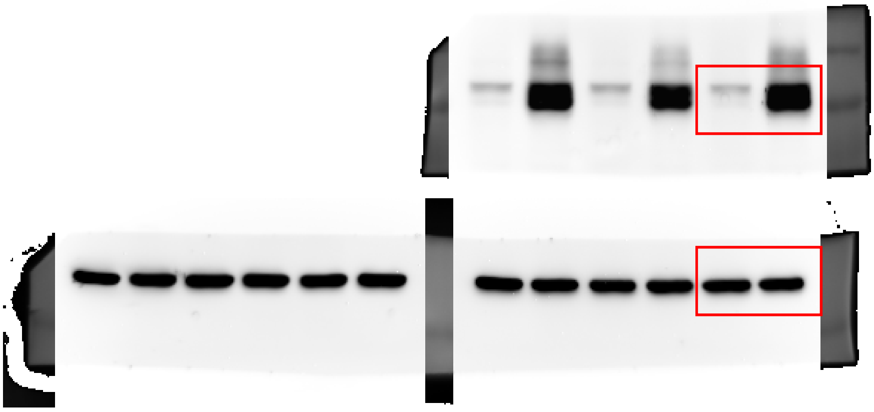

Fig. 6H

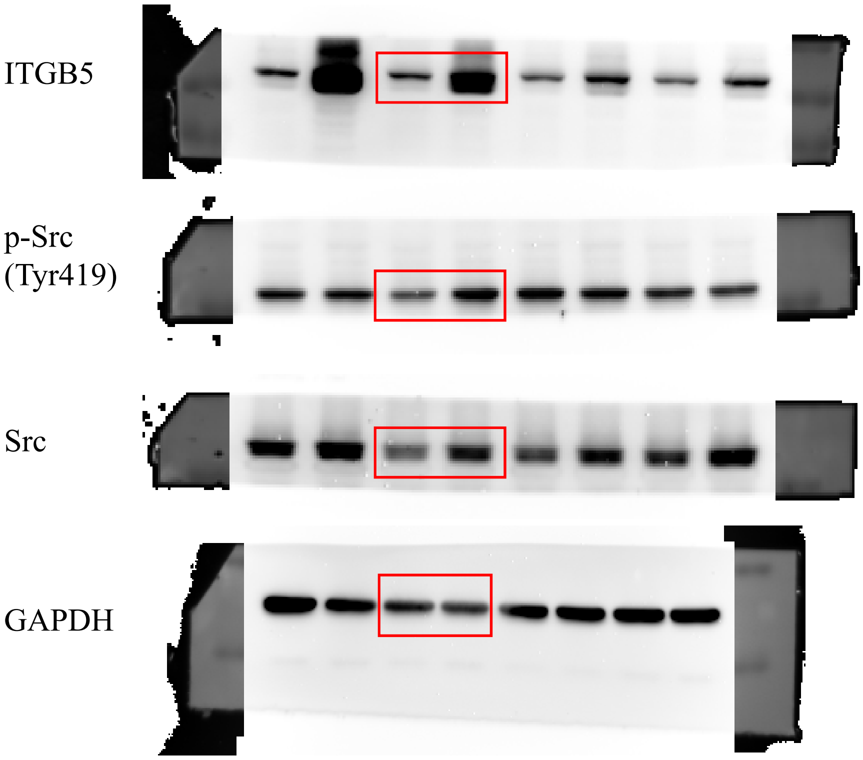

Fig. 7A

ENAH

p-AKT
(Ser473)

AKT

GAPDH

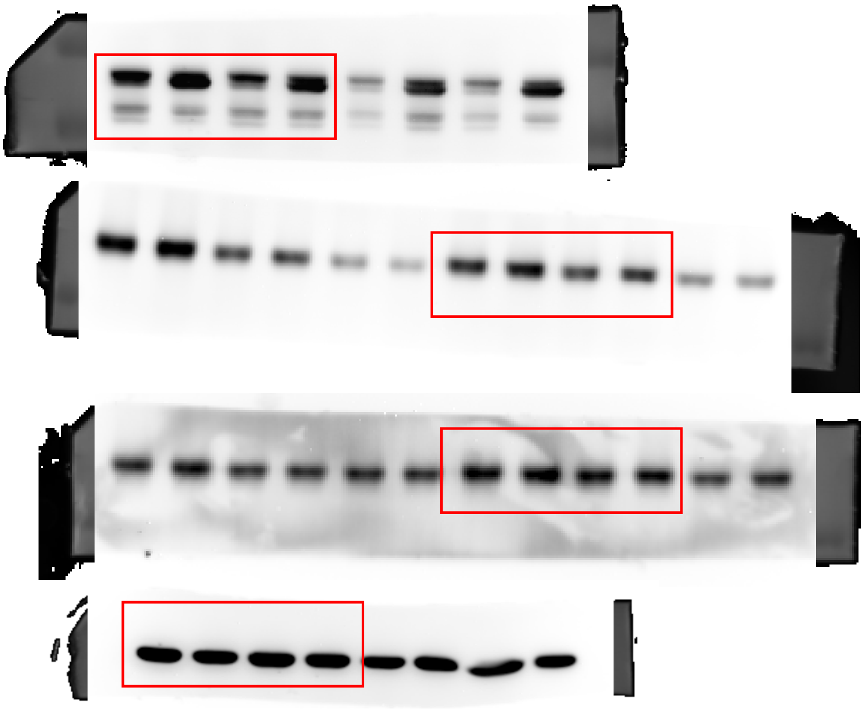

Fig. 7B

ENAH

p-AKT
(Ser473)

AKT

GAPDH

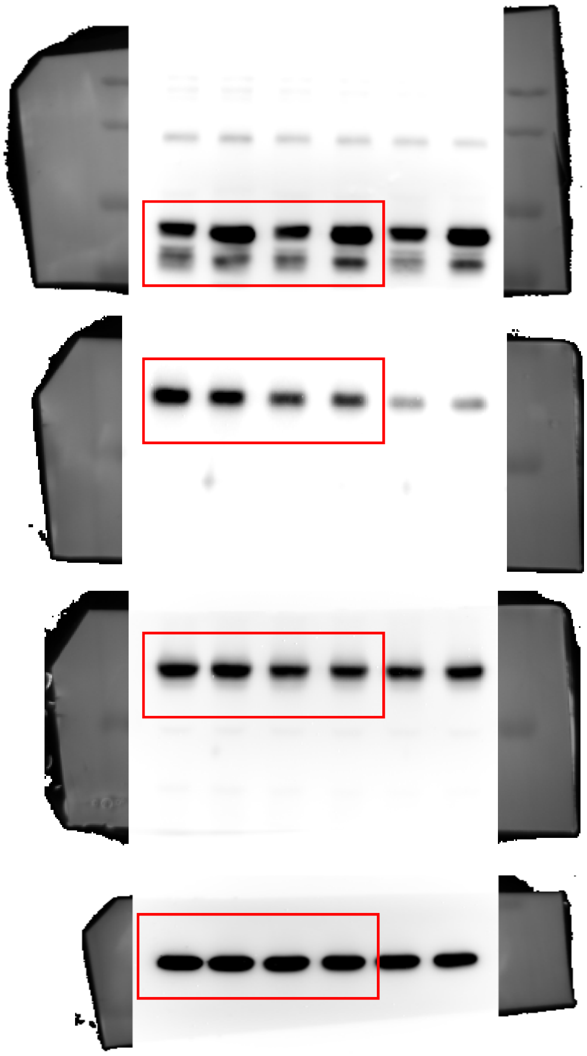

Fig. 7C

ENAH

p-AKT (Ser473)

AKT

GAPDH

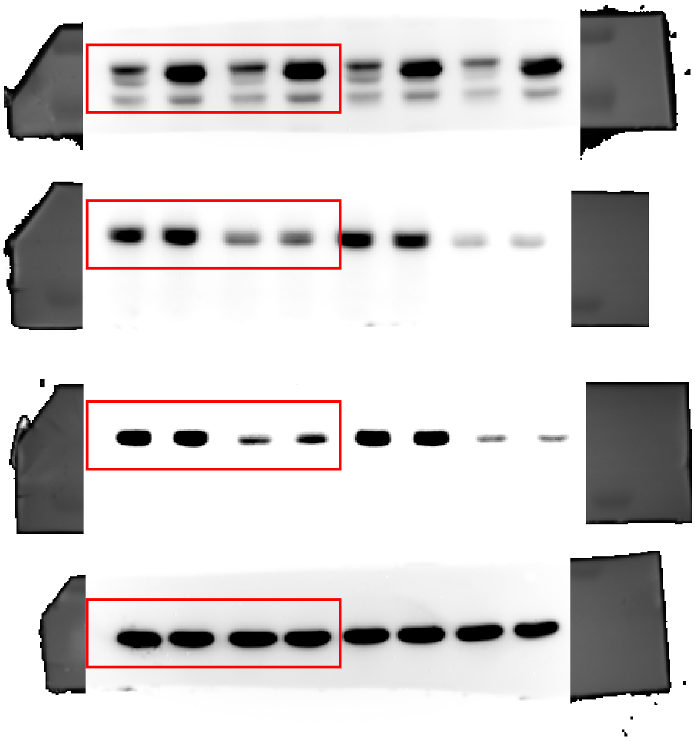

Fig. 7D

ENAH

ITGB5

p-Src (Tyr419)

Src

GAPDH

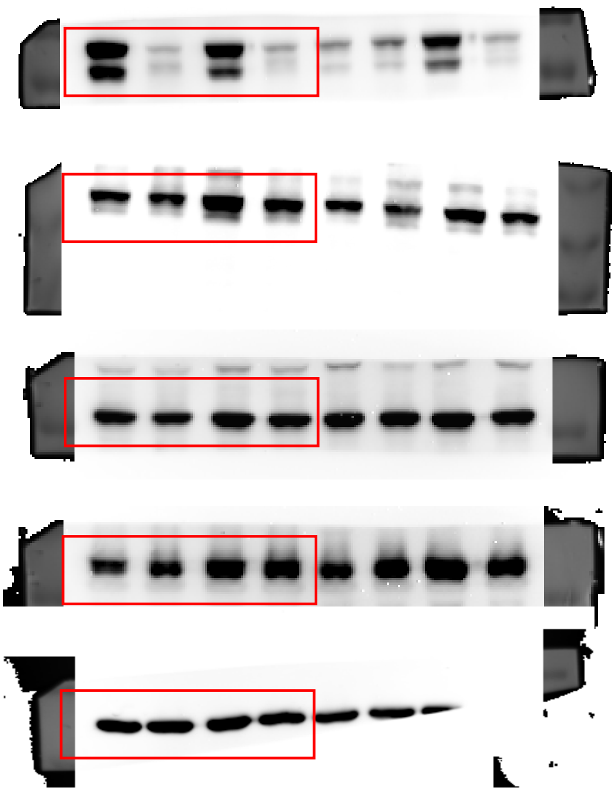

Fig. 7E

ENAH

ITGB5

p-Src (Tyr419)

Src

GAPDH

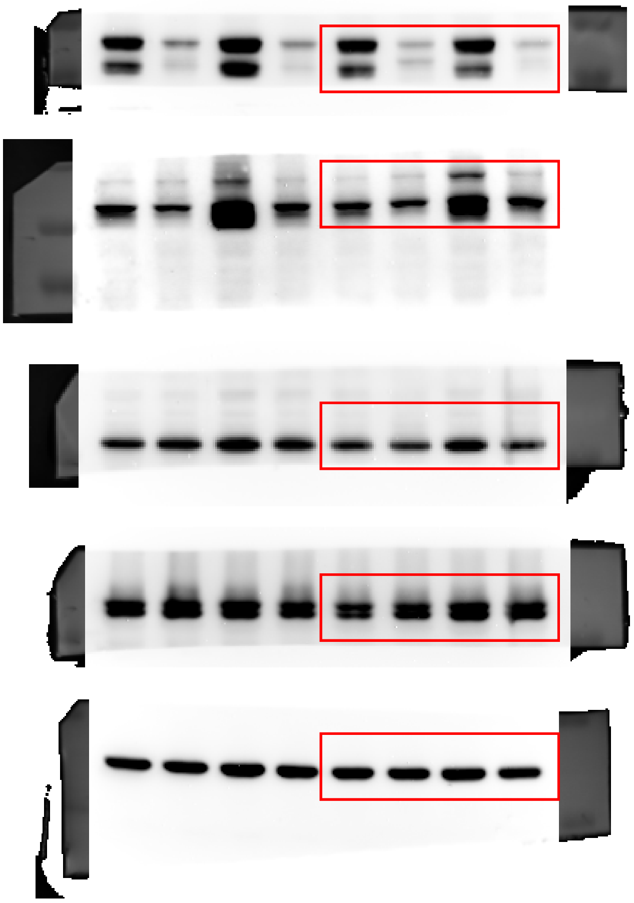

Fig. 7F

ENAH

ITGB5

p-Src (Tyr419)

Src

GAPDH

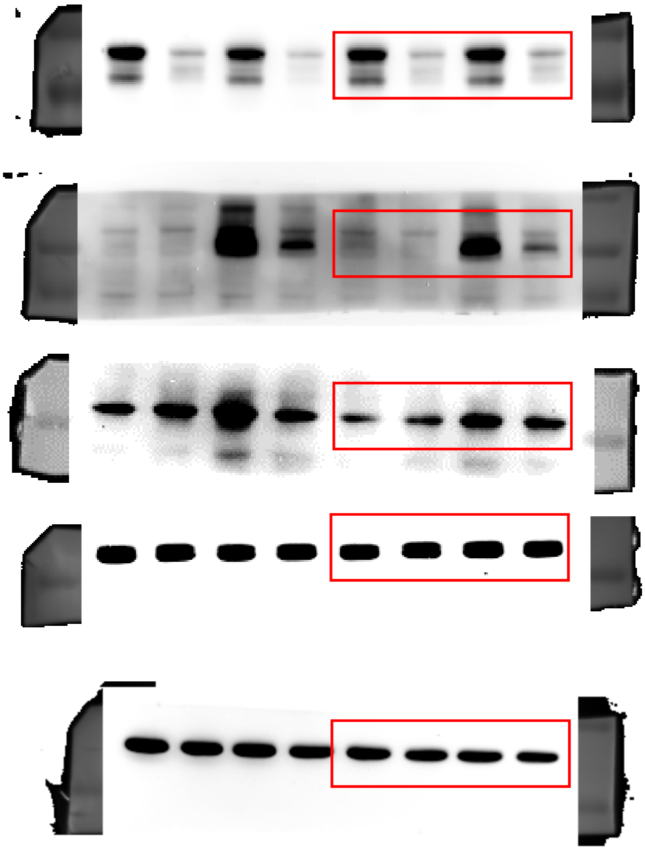

Fig. S3B ENAH

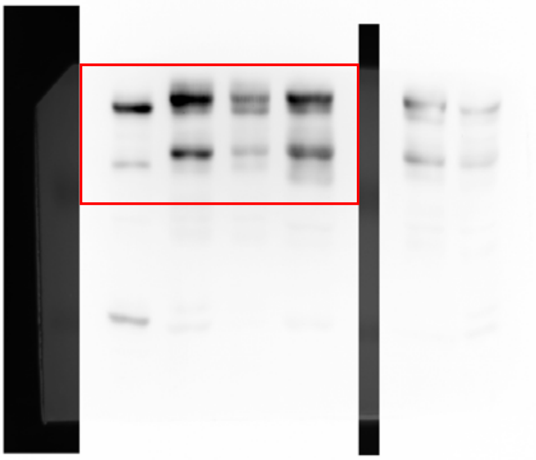

VCP

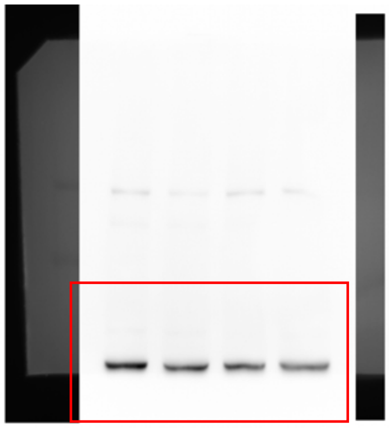

Fig. S3C (left panel) ENAH

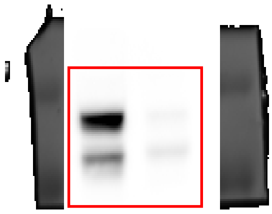

GAPDH

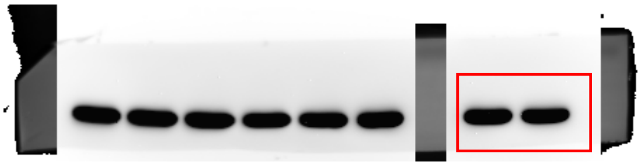

Fig. S3C (right panel) ENAH

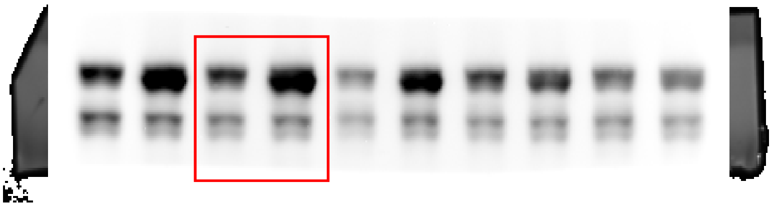

GAPDH

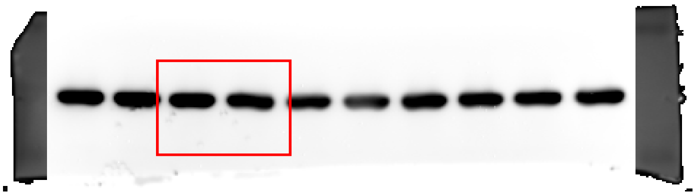

Fig. S4A ENAH

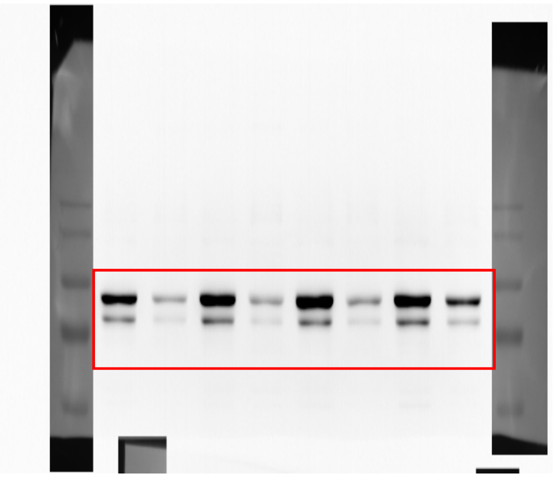

GAPDH

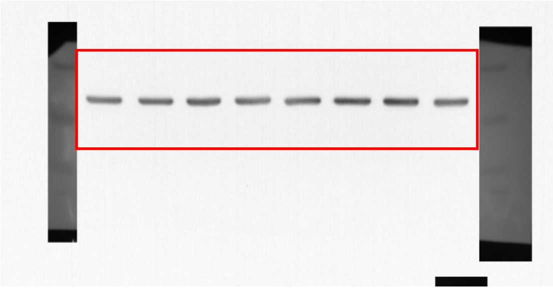

Fig. S4B ENAH

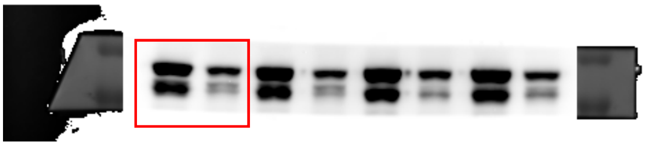

GAPDH

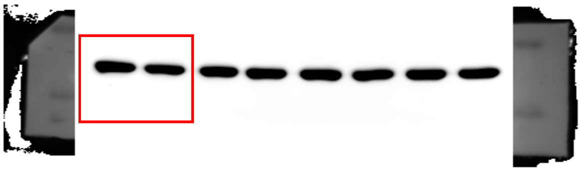

Fig. S4C ENAH

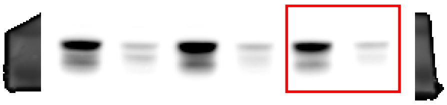

GAPDH

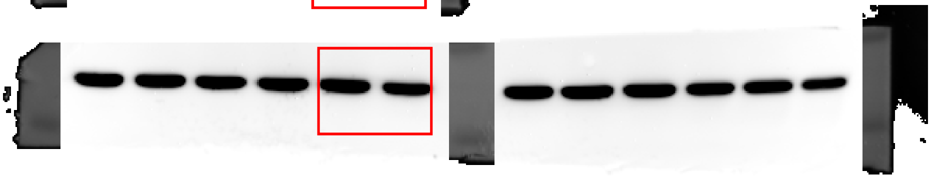

Fig. S4D ENAH

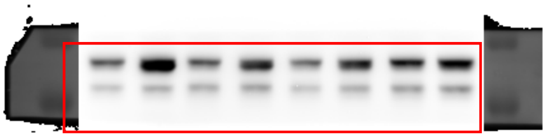

GAPDH

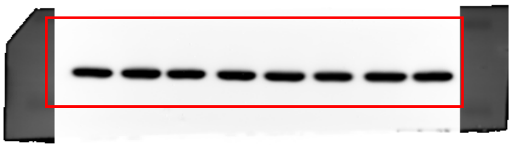

Fig. S4E ENAH

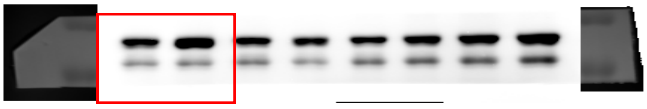

GAPDH

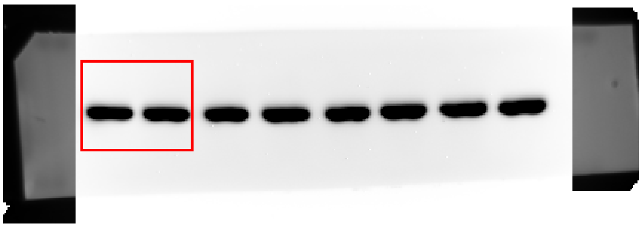

Fig. S4F ENAH

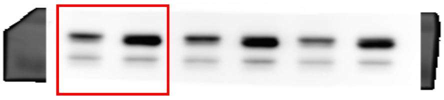

GAPDH

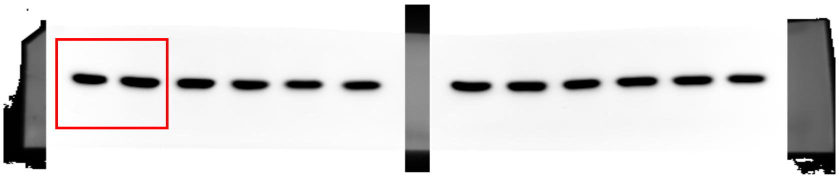

Fig. S5A (left panel)

ENAH

p-AKT (Ser473)

AKT

GAPDH

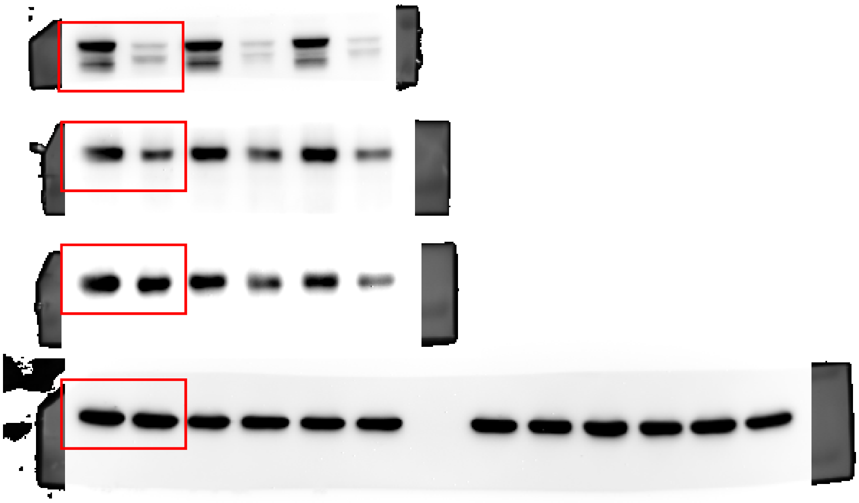

Fig. S5A (right panel)

ENAH

p-AKT (Ser473)

AKT

GAPDH

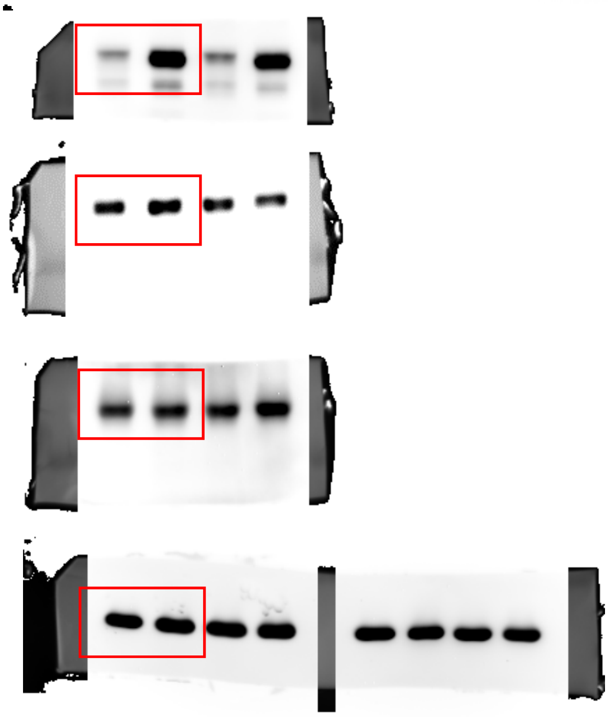

Fig. S5B

ENAH

p-AKT (Ser473)

AKT

GAPDH

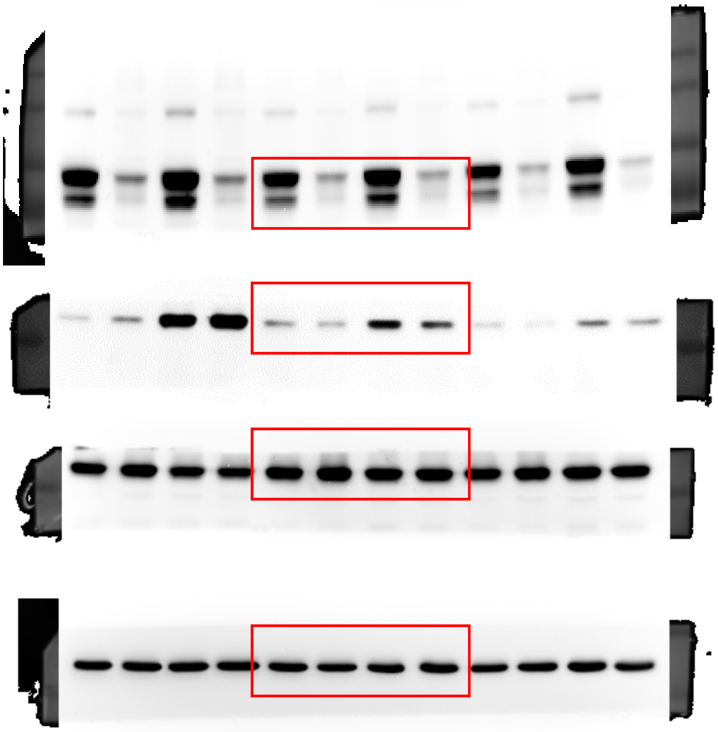

Fig. S7C (left panel)

ENAH

ITGB5

p-Src (Tyr419)

Src

GAPDH

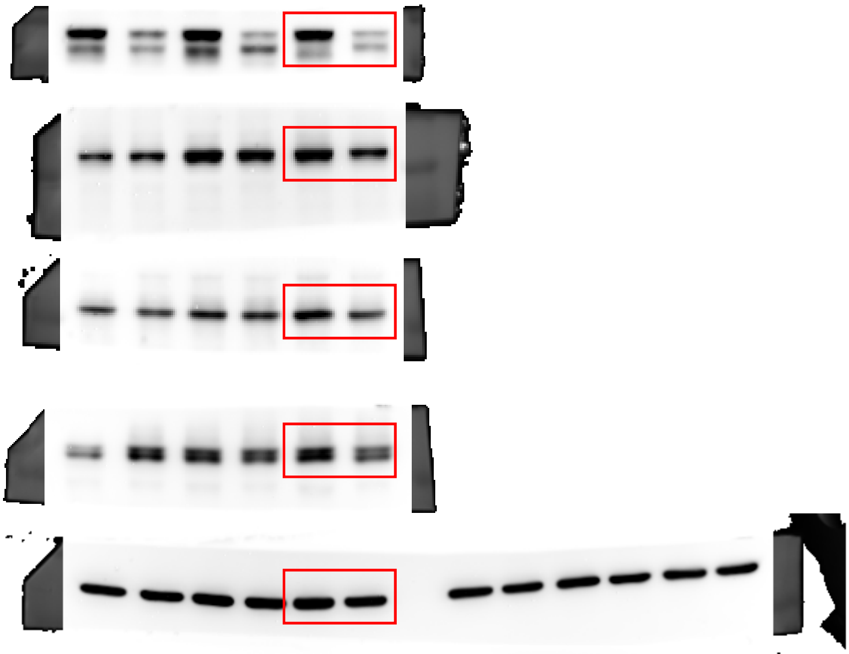

Fig. S7C (right panel)

ENAH

ITGB5

p-Src (Tyr419)

Src

GAPDH

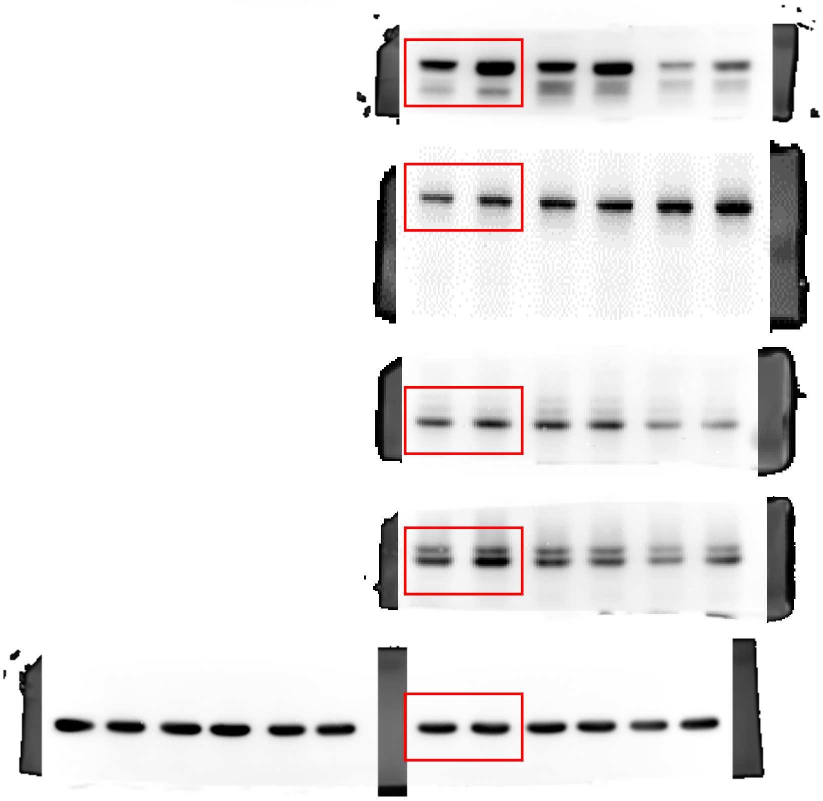

Fig. S7D

ITGB5

GAPDH

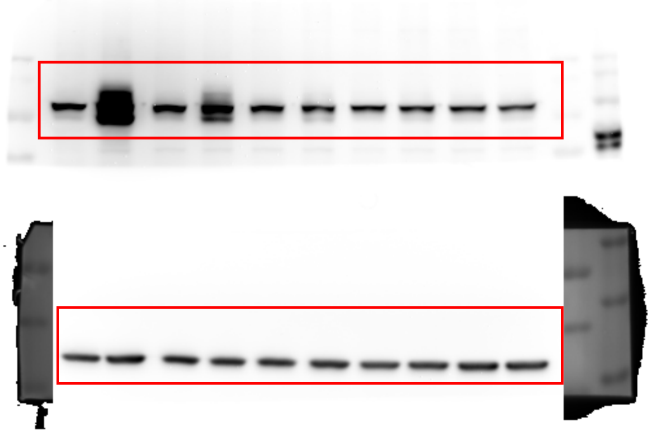

Fig. S7E ITGB5

GAPDH

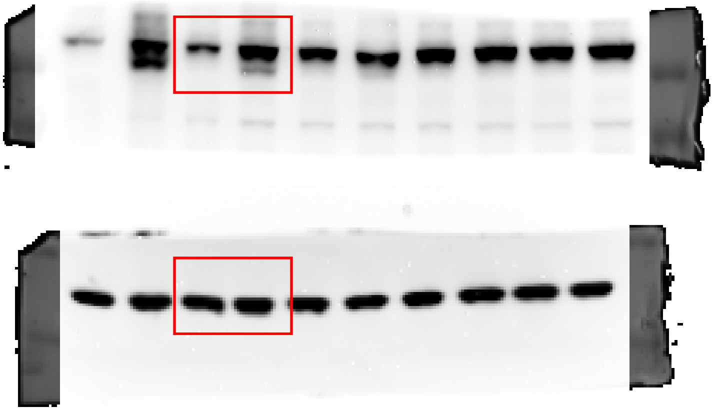

Fig. S7F ITGB5

GAPDH

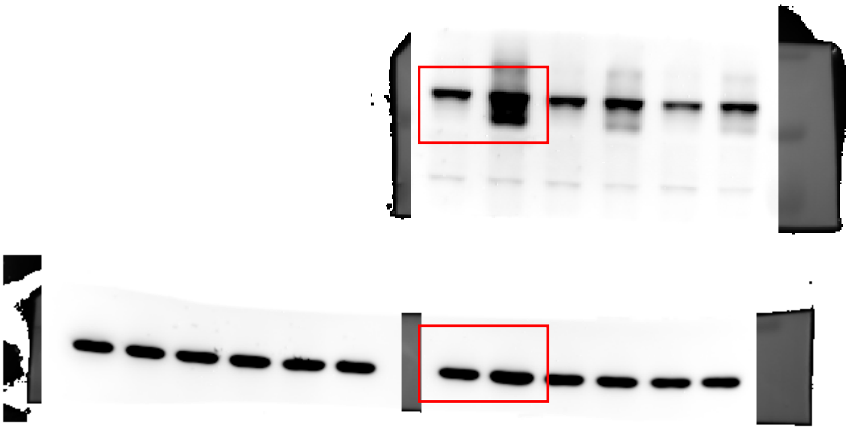

Fig. S7G ITGB5

p-Src
(Tyr419)

Src

GAPDH

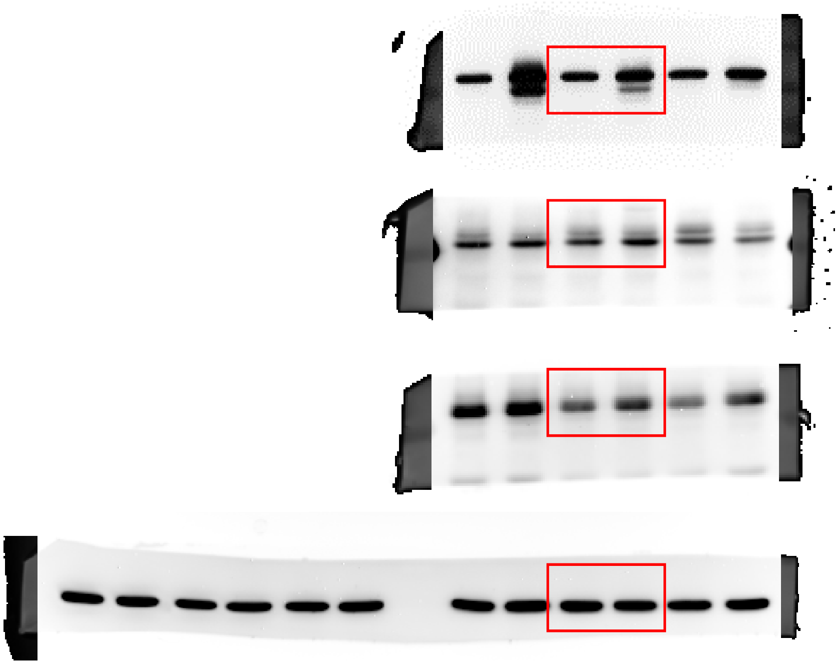

Supplement: Supplementary file 12 — Supplementary material 12: Supplementary Material: Original immunoblots in figures [file 11658_2024_651_MOESM12_ESM.pdf]
